# Distinct Features and Functions of Systemic and Mucosal Humoral Immunity Among SARS-CoV-2 Convalescent Individuals

**DOI:** 10.3389/fimmu.2020.618685

**Published:** 2021-01-28

**Authors:** Savannah E. Butler, Andrew R. Crowley, Harini Natarajan, Shiwei Xu, Joshua A. Weiner, Carly A. Bobak, Daniel E. Mattox, Jiwon Lee, Wendy Wieland-Alter, Ruth I. Connor, Peter F. Wright, Margaret E. Ackerman

**Affiliations:** ^1^ Department of Microbiology and Immunology, Geisel School of Medicine at Dartmouth, Dartmouth College, Hanover, NH, United States; ^2^ Program in Quantitative and Biology Sciences, Dartmouth College, Hanover, NH, United States; ^3^ Thayer School of Engineering, Dartmouth College, Hanover, NH, United States; ^4^ Department of Computer Science, Dartmouth College, Hanover, NH, United States; ^5^ Department of Pediatrics, Geisel School of Medicine at Dartmouth, Dartmouth-Hitchcock Medical Center, Lebanon, NH, United States

**Keywords:** antibody, mucosal immunity, IgA, IgG, neutralization, SARS-CoV-2, COVID-19, systems serology

## Abstract

Understanding humoral immune responses to SARS-CoV-2 infection will play a critical role in the development of vaccines and antibody-based interventions. We report systemic and mucosal antibody responses in convalescent individuals who experienced varying severity of disease. Whereas assessment of neutralization and antibody-mediated effector functions revealed polyfunctional antibody responses in serum, only robust neutralization and phagocytosis were apparent in nasal wash samples. Serum neutralization and effector functions correlated with systemic SARS-CoV-2-specific IgG response magnitude, while mucosal neutralization was associated with nasal SARS-CoV-2-specific IgA. Antibody depletion experiments support the mechanistic relevance of these correlations. Associations between nasal IgA responses, virus neutralization at the mucosa, and less severe disease suggest the importance of assessing mucosal immunity in larger natural infection cohorts. Further characterization of antibody responses at the portal of entry may define their ability to contribute to protection from infection or reduced risk of hospitalization, informing public health assessment strategies and vaccine development efforts.

## Introduction

Since its emergence in late 2019 in China’s Hubei province, SARS-CoV-2, the human coronavirus (CoV) causing COVID-19 disease, has spread rapidly. Formal designation as a pandemic followed in March of 2020, making understanding the health implications of infection and the development of effective interventions a global priority. To this end, studies of immune responses to endemic, other pathogenic CoV strains, and SARS-CoV-2 following infection will contribute to our understanding of how antibodies (Abs) might provide protection from infection or severe disease, or alternatively, contribute to disease pathology.

Due to the critical role of the CoV spike (S) protein in viral entry, Abs targeting S, particularly in the receptor binding domain (RBD), have demonstrated their ability to neutralize the infectivity of CoVs (1–5) including SARS-CoV-2 (6–8). Studies of monoclonal Abs isolated from SARS-CoV-2-infected individuals have shown potent anti-viral effects *in vitro* and protected against viral challenge in mouse, hamster, and nonhuman primate models (9–19), motivating monoclonal antibody-based therapies. Similarly, polyclonal Ab responses in convalescent and vaccinated hamsters and macaques have demonstrated protective efficacy in challenge experiments (20–24). Collectively, these studies establish firm proof of principle for Ab-mediated protection and provide a rationale for the passive transfer of polyclonal serum Abs from recovered individuals as a clinical intervention (25–29).

While information on systemic immunity to SARS-CoV-2 continues to rapidly accrue (30–32), considerable uncertainty still surrounds the role of mucosal immunity to the virus within the respiratory tract, the primary site of SARS-CoV-2 infection and replication (33). Because immune responses can exhibit striking compartmentalization, mucosal immunity induced by natural infection and candidate vaccines administered parenterally may be quite divergent. Indeed, the lack of mucosally-targeted vaccines that have advanced to clinical trials is of some concern (34). While it appears that strong systemic IgG responses can protect against or reduce infection in the lower respiratory tract in animal models (21–24), it may be that Abs in the upper respiratory tract are needed to substantially limit virus replication at this site (34).

Indeed, early work to delineate Ab responses to human (35) and animal CoV (36–38) suggests that the induction of mucosal Ab is a key component in reducing viral shedding after infection and may mediate protective immunity following re-exposure. Studies of mucosally-targeted SARS-CoV-1 vaccines in animal models have identified that virus-specific mucosal IgA mediates protection against subsequent exposure to wild-type CoV (39, 40). These observations suggest that strategies to prevent SARS-CoV-2 infection will benefit from effective induction of not only robust systemic immunity, but also functional mucosal immunity to prevent or limit infection at the portal of entry (41). Thus, robust preexisting or induced mucosal immunity could reduce the risk of transmission within the community by decreasing the level or duration of viral shedding from infected individuals.

## Methods

### Human Subjects

A total of 35 individuals were studied (**Supplemental Table 1**); 20 who had recovered from COVID-19 (age range: 18–77, mean: 53 years) and 15 naïve control subjects (age range: 22–66, mean: 40 years). Infection with SARS-CoV-2 was confirmed by PCR of nasopharyngeal swab for all COVID-19 patients. Study subjects included both males (17) and females (18). Disease severity among COVID-19 subjects ranged from mild (4) to moderate (12) and severe (4). Classification of mild and moderate disease was based individuals self-reporting whether or not their symptoms influenced activities of daily life, while a designation of severe disease was made on the basis of hospitalization for COVID-19. Serum, nasal wash, and stool samples were collected from each donor approximately 1 month after symptom onset (range: 19–67 days, mean: 38 days).

Primary neutrophils used in functional assays were purified from deidentified blood samples from healthy male and female donors over the age of 18 years. All research involving human subjects was approved by the Dartmouth College and Dartmouth-Hitchcock Medical Center Committee for the Protection of Human Subjects (Institutional Review Board) and written informed consent was obtained from all participants.

### Antigen and Fc Receptor Expression and Purification

Prefusion-stabilized, trimer-forming spike protomers (S-2P) of SARS-CoV-2, closely related and/or epidemic strains (SARS-CoV-1, WIV1, and MERS), and endemic coronaviruses (229E, OC43, NL63, and HKU1), and the receptor-binding domain of SARS-CoV-2 fused to a monomeric form of the human IgG4 Fc region were transiently expressed in either Expi 293 or Freestyle 293-F cells, and purified *via* affinity chromatography according to the manufacturers’ protocols (**Supplemental Tables 2** and **3**). Human FcgR were expressed and purified as described previously (42).

### Fc Array Assay

CoV and control antigens, including S trimers, S subdomains (*i.e.*, S1 and S2), and other viral proteins from SARS-CoV-2 as well as S and S subdomains from SARS CoV-1, MERS, HKU1, OC43, NL63, 229E, and WIV1 (**Supplemental Table 2**) and influenza HA and herpes simplex virus (HSV) gE proteins were covalently coupled to Luminex MagPlex magnetic microspheres using a two-step carbodiimide chemistry as previously described (43). Biotinylated SARS-CoV-2 fusion peptide was captured on neutravidin-coupled microspheres. Pooled polyclonal serum IgG (IVIG), CR3022, a SARS CoV-1-specific monoclonal Ab that cross-reacts with SARS-CoV-2 S (11), and VRC01, an HIV-specific monoclonal Ab, were used as controls to define the antigenicity profiles. The optimal dilution of serum was determined in pilot experiments in which a subset of samples was titrated. Test concentrations for serum ranged from 1:250 to 1:5000 and varied per detection reagent. Nasal wash and stool samples were assayed at a 1:10 dilution. Isotypes and subclasses of antigen-specific Abs were detected using R-phycoerythrin (PE) conjugated secondary Abs and by FcRs tetramers (**Supplemental Table 4**) as previously described (44, 45). A FlexMap 3D array reader detected the beads and measured PE fluorescence used to calculate the Median Fluorescence Intensity (MFI).

### Neutralization Assay

Samples of serum and nasal wash from SARS-CoV-2 convalescent and naïve donors were tested in microneutralization assays using a VSV-SARS-CoV pseudovirus system (46). In brief, samples were serially diluted 2-fold (1:50-1:3200 for serum; 1:4-1:256 for nasal wash) and incubated with a standardized concentration of SARS-CoV-2 pseudovirus for 1 h at 37°C followed by addition to duplicate wells of 293T-ACE2-expressing target cells (Integral Molecular, Philadelphia PA) in a final volume of 100 μl per well. Plates were incubated at 37°C for 18–24 h, after which luciferase activity was measured using the Bright-Glo system (Promega, Madison WI) in a Bio-Tek II plate reader. Results were quantified relative to controls and data expressed as 60% neutralization titers.

### Phagocytosis Assays

Assays of Ab-dependent phagocytosis by monocytes (ADCP) and neutrophils (ADNP) were performed essentially as described (47–49). Briefly, 1 µm yellow-green fluorescent microspheres (Thermo, F8813) were covalently conjugated with recombinant RBD and incubated for 3 h with dilute serum or nasal wash specimens and either the human monocytic THP-1 cell line (ATCC, TIB-202), or with freshly-isolated primary neutrophils. After pelleting, washing, and fixing, phagocytic scores were quantified as the product of the percentage of cells that phagocytosed one or more fluorescent beads and the median fluorescent intensity of this population as measured by flow cytometry with a MACSQuant Analyzer (Miltenyi Biotec). ADCP assays were performed in duplicate with high correspondence between results presented here and the replicate run. ADNP assays were performed in biological replicate using neutrophils purified from two different healthy donors for which results were averaged. A subset of neutrophils was stained with CD66b-APC (BioLegend G10F5) and PI (Biotium 41007) to determine the purity and viability of the isolated cellular fraction. CR3022 and VRC01 were used as positive and negative controls, respectively. Wells containing no Ab were used to define the level of Ab-independent phagocytosis.

### CD16 Reporter Assay

The ADCC potential of the specimens was measured using a Jurkat Lucia NFAT cell line (Invivogen, jktl-nfat-cd16), cultured according to the manufacturer’s recommendations, in which engagement of FcγR3a (CD16) on the cell surface leads to the secretion of luciferase. One day prior to running the assay, a high binding 96 well plate was coated with 1 µg/ml SARS-CoV-2 RBD at 4°C overnight. Plates were then washed with PBS + 0.1% Tween20 and blocked at room temperature for 1 h with PBS + 2.5% BSA. After washing, dilute serum or nasal wash sample and 100,000 cells/well in growth medium lacking antibiotics were cultured at 37°C for 24 h in a 200 µl volume. The following day, 25 µl of supernatant was drawn from each well and transferred to an opaque, white 96 well plate, to which 75 µl of QuantiLuc substrate was added and luminescence immediately read on a SpectraMax Paradigm plate reader (Molecular Devices) using 1 s of integration time. The reported values are the mean of three kinetic reads taken at 0, 2.5, and 5 min. Negative control wells substituted assay medium for sample while 1x cell stimulation cocktail (Thermo, 00-4970-93) plus an additional 2 μg/ml ionomycin were used to induce expression of the transgene as a positive control.

### Complement Deposition Assay

Antibody-dependent complement deposition (ADCD) was quantified essentially as previously described (50). In brief, serum and nasal samples were heat-inactivated at 56°C for 30 min prior to a 2 h incubation at a dilution of 1:20 at RT with multiplex assay microspheres. After washing, each sample was incubated with human complement serum (Sigma, S1764) at a concentration of 1:50 at RT with shaking for 1 h. Samples were washed, sonicated, and incubated with murine anti-C3b (Cedarlane #CL7636AP) at RT for 1 h followed by anti-mouse IgG1-PE secondary Ab (Southern Biotech #1070-09) at RT for 30 min. After a final wash and sonication, samples were resuspended in Luminex sheath fluid and complement deposition was determined on a MAGPIX (Luminex Corp) instrument to define the MFI. Assays performed without Ab and with heat-inactivated human complement serum were used as negative controls.

### Immunoglobulin Depletion

The antibody isotypes present in nasal wash samples were selectively depleted using resins targeting IgG (CaptureSelect IgG CH1 affinity matrix, Thermo, 194320005), IgA (CaptureSelect IgA affinity matrix, Thermo, 194288005), or IgM (POROS CaptureSelect IgM affinity matrix, Thermo, 195289005). Microcentrifuge spin columns (Thermo, 69705) were loaded with 50 µl of suspended resin, followed by equilibration of the resin by passing PBS through the column. Nasal wash samples were diluted 1:10 in PBS and 150 µl were added to the column and incubated with end-over-end mixing for 5 min at room temperature. The remaining volume of the diluted sample was saved for use as the mock-depleted control. The isotype-depleted flow through was collected in a clean microcentrifuge tube and the columns were regenerated with 100 mM glycine (pH 3.0) and equilibrated before reuse. The extent of on- and off-target depletion of IgG, IgA, and IgM was measured using the Fc array assay described previously. The depleted samples were then evaluated in neutralization and phagocytosis assays as described above.

### Data Analysis and Visualization

Basic analysis and visualization were performed using GraphPad Prism. Heatmaps, correlation plots, and boxplots were made in R (version 3.6.1 (51), supported by R packages pheatmap (52), corrplot (53), and ggplot2 (54)). Hierarchical clustering was used to cluster and visualize data using Manhattan or Euclidean distance. Fc Array features were filtered by elimination of measurements for which >25% of the samples exhibited signal within 10 standard deviations (SD) of the technical blank. Fc array features were log transformed, then scaled and centered by their standard deviation from the mean (z-score). Based on data distributions and the limited number of individuals who experienced severe disease, a student’s two-tailed t-test with Welch’s correction with a cutoff of p = 0.05 was used to define features different between groups and with the exception of depletion studies, Pearson correlation coefficients were calculated to define relationships between features. Non-parametric tests yielded qualitatively similar results; parametric test results are presented due to their interpretability and power advantage. All comparison results should be considered in the context of limitations of small cohort size. For depletion studies, Spearman correlation coefficients and two-tailed p values were determined for the relationship between extent of depletion and extent of decrease in neutralization and phagocytosis, according to data distributions and the lack of an expectation of a monotonic linear relationship. Increases were considered to be within assay noise and set to zero. Cohort characteristic, Fc Array, and functional assay data is available at https://github.com/AckermanLab/Butler_et_al_COVID_2020.

## Results

### Systemic and Mucosal SARS CoV-2 Specific Ab Response Features

To characterize and compare the systemic and mucosal humoral immune responses to SARS-CoV-2 and better understand the relationships between antibody features and functions within serum, nasal wash, and stool samples, we turned to systems serology (55). This technique utilizes high-throughput, multidimensional biophysical profiling of antibody response features, cell-based assays of Ab neutralization and effector functions, and machine learning as a means to discover mechanistically meaningful signatures of Ab-mediated protection and activity (56).

Serum, nasal wash, and stool samples were collected approximately 1 month after initial clinical presentation from 20 subjects who tested positive for SARS CoV-2 by qPCR, and from 15 SARS-CoV-2 naïve subjects (**Supplemental Table 1**). Recruitment of a larger cohort of convalescent subjects was limited by the low case burden in the area where these samples were obtained. Antibody responses to SARS-CoV-2 were evaluated using an Fc array (44, 45) to characterize isotypes, subclasses, and Fc receptor (FcR) binding across Abs specific to a panel of SARS-CoV-2 antigens. This panel included stabilized trimeric spike protein (S-2P), subunits (i.e. S1, S2), and receptor binding domain (RBD) forms, nucleocapsid (N) protein, and the fusion peptide. S proteins from four other endemic CoV strains (OC43, HKU1, 229E, and NL63) and two non-CoV control antigens (influenza HA and herpes simplex virus gE), were also evaluated.

**Figure 1:**
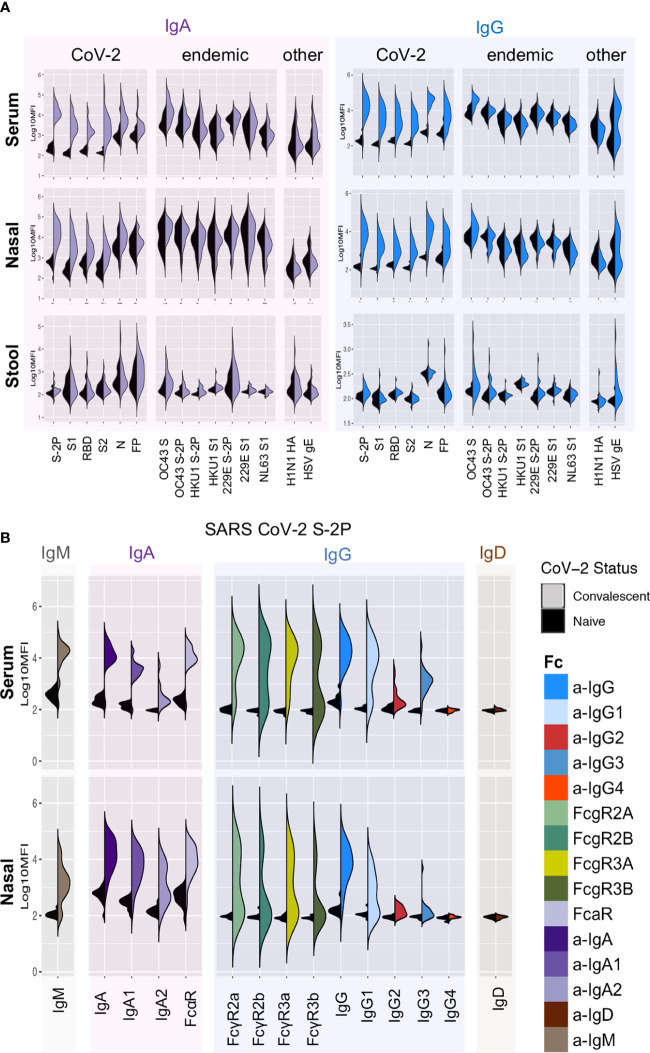
Systemic and mucosal Ab responses. **(A)** Fc array characterization of IgA (left) and IgG (right) responses against a panel of SARS-CoV-2, other CoV, and control antigens in serum (top), nasal wash (middle), and stool (bottom) from convalescent (colored) and naïve (black) donors. **(B)** Isotypes, subclasses, and FcR binding of Abs to stabilized SARS CoV-2 S (S-2P) in serum (top) and nasal wash (bottom).

SARS-CoV-2-specific Ab responses were observed in COVID-19-convalescent, but not naïve, donor sera, nasal wash, and stool (**Figure 1A**). Ab responses in serum and nasal wash samples were examined by measuring levels of Ab isotypes and subclasses, and by defining binding to diverse FcRs (**Figure 1B**, **Supplemental Figures 1–6**). SARS CoV-2-specific IgG1, IgG2, IgG3, IgA, IgM, and immunoglobulin (Ig) able to ligate FcRs (FcαR, FcγR) were observed among samples from convalescent donors but not naïve subjects. In contrast to the robust responses apparent in serum and nasal samples, limited SARS CoV-2 specific Ig was detected in stool samples (**Figure 1A**, **Supplemental Figure 7**). Robust responses to stabilized spike (S-2P) and N were observed, as were responses to functionally relevant RBD and fusion peptide domains.

Systematic analysis of the magnitude and statistical confidence of differences in Ab present in samples from convalescent versus naïve donors indicated elevated IgG, IgA, and IgM responses across diverse SARS CoV-2 antigens as well as a number of antigens from endemic and related beta-CoV (**Figure 4A**, **Supplemental Figure 8**). Among these, elevated levels of OC43 (29.7% S amino acid identity) S-specific IgG and IgA in serum were particularly notable (unpaired two-tailed t-test with Welch’s correction; p < 0.0001 and p = 0.011 respectively). This apparent boosting of responses to endemic CoV occurred more widely among IgG1 and IgG3 subclasses (**Supplemental Figure 1**) and was also apparent in nasal and stool samples (**Supplemental Figures 4, 7**).

### Neutralization Activity of Systemic and Mucosal Ab

**Figure 2:**
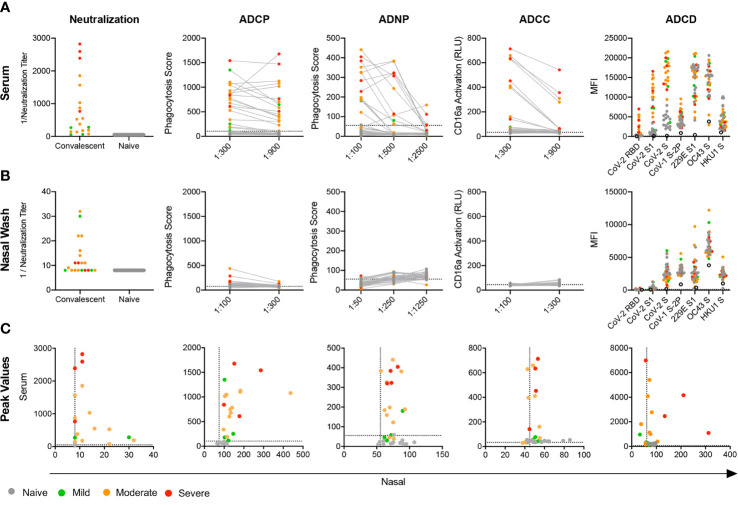
Mucosal and systemic Ab functions. **(A, B)** Functional activity of serum **(A, B)** nasal wash subject samples in a panel of neutralization and effector function assays including antibody-dependent phagocytosis by monocytes (ADCP) and neutrophils (ADNP), action of FcγRIIIA (ADCC), and complement cascade C3b deposition (ADCD). **(C)** Scatterplots of serum versus nasal wash sample activities observed for each subject for viral neutralization and RBD-specific Ab effector functions. Titer is plotted for neutralization data and peak activity is plotted for effector functions. Infection status and disease severity is indicated in color. Limit of detection (neutralization) or values observed for no Ab controls (ADCP, ADNP, ADCC) are indicated with dotted lines. No Ab controls for ADCD are indicated with the hollow black circle.

Given evidence of robust humoral responses in systemic and mucosal samples, we next sought to determine the neutralization potency of serum and nasal wash samples using a luciferase-based SARS-CoV-2 pseudovirus assay (46). Consistent with other studies (57), elevated serum neutralization activity was observed for hospitalized subjects who experienced severe, as compared to non-severe (i.e. mild and moderate cases), disease (unpaired two-tailed t-test with Welch’s correction, p = 0.034) (**Figure 2A**). In contrast to observations made in serum, nasal samples from subjects with severe disease showed little to no viral neutralization, whereas subjects with elevated mucosal neutralization activity tended to report mild symptoms that did not influence activities in their daily lives or moderate symptoms that did influence activities but did not require hospitalization (**Figure 2B**). Interestingly, robust nasal and serum neutralization activities were not co-induced (**Figure 2C**).

### Effector Functions of Systemic and Mucosal Ab

Beyond neutralization, little is known about the antiviral functions of systemic and mucosal Abs in COVID-19 convalescent donors. We characterized the antiviral effector functions of Abs in serum and nasal samples by evaluating Ab-mediated phagocytosis, NK cell receptor ligation and complement activation induced by RBD-specific antibodies. Serum from most convalescent subjects readily promoted phagocytosis mediated by monocyte (ADCP) and neutrophil (ADNP) effector cells (**Figure 2A**). While nasal wash samples were far less capable of driving functional activity, several subjects exhibited nasal Ab responses able to elicit phagocytosis in monocytes (**Figure 2B**). Serum from these subjects also tended to generate a strong phagocytic response (**Figure 2C**). Across phagocytosis, NK cell FcγR3a receptor ligation (ADCC), and deposition of complement cascade protein C3b (ADCD), a pattern of elevated Ab effector function emerged among subjects who experienced moderate or severe disease; those who experienced mild disease generated little activity. In contrast to serum, but consistent with the lower relative levels of IgG detected, nasal wash initiated only limited ADNP, ADCC, and ADCD, whereas monocyte phagocytosis was apparent (**Figure 2B**), and directly correlated with ADCP activity in serum (**Figure 2C**).

### Functional Correlates in Serum and Mucosal Abs

**Figure 3:**
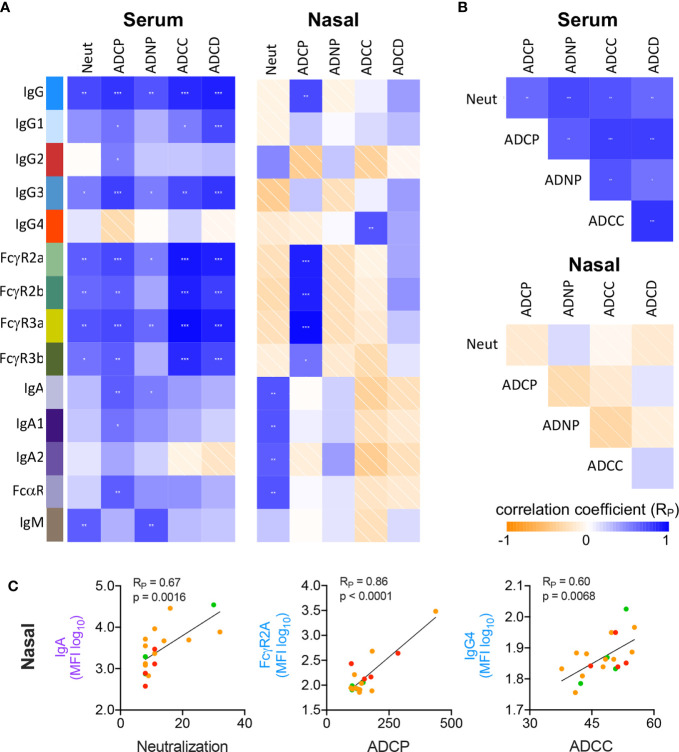
Correlative relationships between RBD-specific Ab features and functions. **(A)** Correlations observed between RBD-specific Ab features and functions in serum (left) and nasal wash (right) samples. **(B)** Correlations observed between Ab functions observed in serum (top) and nasal wash (bottom). **(C)** Representative scatterplots between highly correlated Ab features and functions in nasal wash samples. p values from unpaired two tailed t-tests with Welch’s correction. Pearson correlation coefficients (R_P_) are shown.

Next, we explored the characteristics of the Abs mediating each effector function by measuring correlations between RBD-specific Ab biophysical features and Ab functions in serum (**Figure 3A**). ADCP, ADNP, and ADCC activities were most strongly correlated with FcγR binding, consistent with their reliance on this class of receptor, but also with levels of IgG1 and IgG3, which ligate FcγR best among human IgG subclasses. Interestingly, IgM positively correlated with both ADNP and neutralization activity. While IgA responses were robustly induced in serum, this isotype was generally more weakly associated with neutralization and effector activities than IgG.

In nasal samples, however, neutralization activity positively correlated with the IgA response (**Figures 3A, C**; R_P_ = 0.67, p = 0.0016). In comparison, and as in serum, ADCP showed significant correlation with total RBD-specific IgG and FcγR-binding RBD-specific Abs (R_P_ = 0.86, p < 0.0001). Strong relationships with the other effector functions, which largely showed low or negligible levels of activity, were generally not observed. While serum Ab functions were significantly correlated with one another, this relationship was not seen in nasal wash samples (**Figure 3B**), attributable to the low activity observed for many functions. Representative scatterplots between individual features and functions show how these activities and characteristics varied by subject according to disease severity (**Figure 3C**).

### Relationships Between Systemic and Mucosal Ab Features

**Figure 4:**
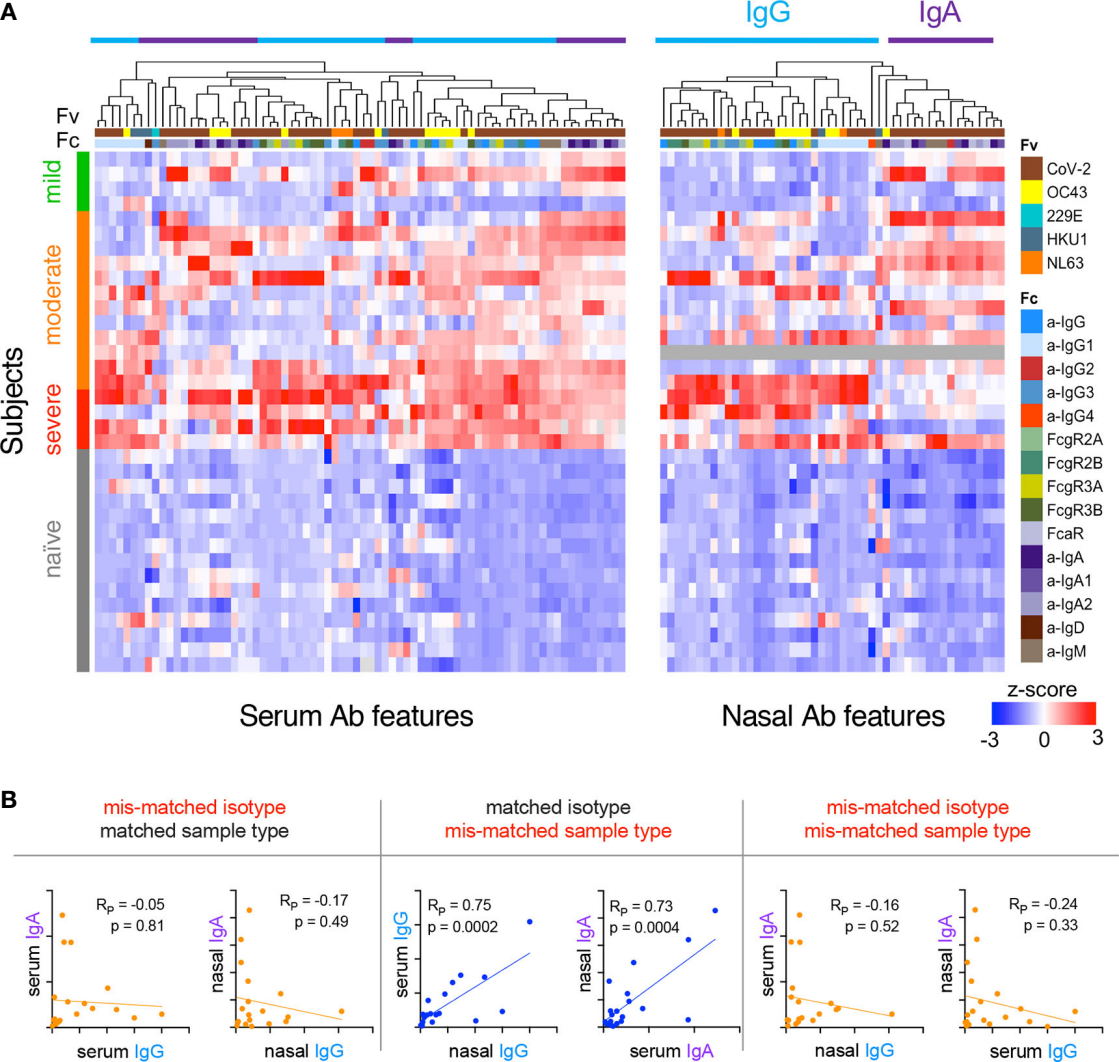
Relationships among subjects and Ab features in serum and nasal wash. **(A)** Heatmap of filtered and hierarchically-clustered Fc array features in serum (left) and nasal wash (right) across subjects with varying infection or disease status. Each row represents an individual donor. Disease severity is shown on the left annotation bar. Each column represents an Fc Array measurement, with antigen specificity (Fv) and Fc characteristics (Fc) are indicated in top color bars. Responses are centered and scaled per feature and the scale range truncated at +/-3 SD. Relatively high responses are indicated in red, and low responses in blue. Color bars at bottom indicate the divergent clusters of IgG-related (blue) and IgA-related (purple) responses observed in nasal wash samples. **(B)** Representative scatter plots of the correlative relationships between IgA and IgG anti-S1 responses in nasal and serum samples. Pearson correlation coefficients (R_P_) and p values of unpaired two-tailed t-tests with Welch’s correction are reported.

Because mucosal and systemic responses can exhibit remarkable divergence (58), we next explored relationships among features of the humoral immune response between these two compartments. Following centering and scaling, hierarchical clustering of Ab features that were significantly increased among convalescent donors (unpaired two-tailed t-test with Welch’s correction, p<0.05, **Supplemental Figure 8**) was performed to define similarities between subjects and features (**Figure 4A**). Elevated IgG responses were apparent in both serum and nasal samples in convalescent donors who experienced severe disease. In contrast, donors with non-severe disease (mild or moderate) appeared to present elevated nasal IgA responses (**Figure 4A**, upper right).

Given this apparent dichotomy between isotypes, correlations between IgG and IgA within and between serum and nasal samples of convalescent donors were defined for a representative S antigen (**Figure 4B**). Serum and nasal IgG were directly correlated, as were serum and nasal IgA. However, no or inverse relationships were observed between IgG and IgA both within and between sample types. When expanding this analysis to all Ab types and specificities elicited among convalescent donors, clear patterns emerged suggesting global biases between IgA and IgG responses (**Supplemental Figure 9A**). For example, in serum, IgG1, IgG3, and FcγR-binding Ab responses correlated well with one another across diverse specificities; IgA, IgA1, IgA2, and FcαR-binding Abs did so too. Overall, correlations between IgG and IgA in serum were more modest, though many specific response features were positively correlated with each other. In contrast, nasal responses showed evidence of a dichotomy between isotypes with a tendency to favor either IgG or IgA across diverse antigen specificities (**Figure 4B**, **Supplemental Figure 9B**).

### Relationships Between Clinical Characteristics and Ab Responses

Though power is limited by the small cohort, we explored how immune responses related to subject characteristics by defining the magnitude and statistical confidence of differences between subjects by age, sex, and disease severity (**Supplemental Figure 10**). Relatively few aspects of the antibody response differed according to sex and age. These differences were considerably less pronounced than those observed to vary according to disease severity, though some exceeded a nominal significance threshold of p = 0.05, and thus may merit follow up in other cohorts.

**Figure 5:**
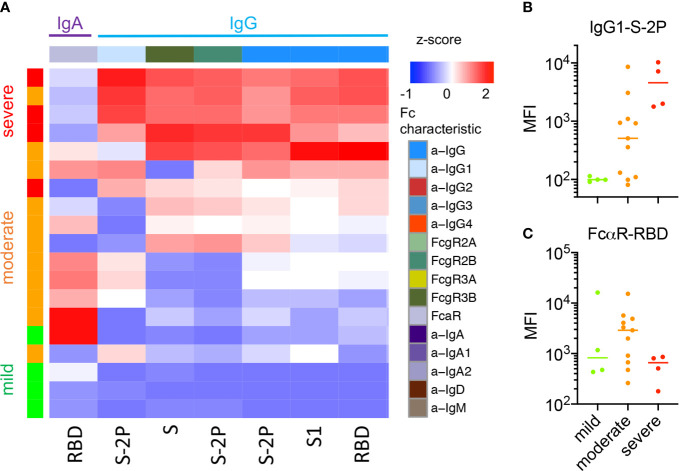
Nasal Ab features associated with disease severity. **(A)** Heatmap of nasal CoV-2-specific Ab features that exhibited statistically significant differences in responses between subjects with severe and non-severe (mild or moderate) disease (unpaired two-sided t-test with Welch’s correction, p < 0.05). **(B, C)**Representative boxplots of nasal features by disease severity across donors who experienced mild, moderate, and severe disease. **(B)** IgG1 specific to S-2P as a representative Ab feature elevated among subjects with severe disease. **(C)** FcαR binding Abs specific to RBD are highest among individuals who recovered from moderate disease as opposed to either mild or severe disease.

In contrast, a greater number of features were more robustly and confidently distinct among individuals according to disease severity, though confidence is limited by the small number of subjects in the cohort who experienced severe disease. In exploratory analyses of serum and nasal wash samples, comparisons were performed to determine which CoV-2-specific Ab features showed differences between individuals experiencing severe versus non-severe (mild or moderate) disease (unpaired two-sided test with Welch’s correction, p < 0.05). As reported in other cohorts (57), a number of IgG-related responses were elevated in serum among individuals who had experienced severe disease (**Supplemental Figure 11**). One feature elevated in those with severe disease, CoV-2-specific antibodies interacting with FcγR3a, was recently reported to also be associated with fatal disease (31). This heightened IgG response among hospitalized donors was also evident in nasal wash samples, whereas RBD-specific antibodies that bound FcαR were significantly elevated in the nasal wash samples of subjects who had experienced mild or moderate disease (unpaired two-sided test with Welch’s correction, p = 0.025) (**Figure 5A**), though this difference was not significant following Bonferroni correction. A number of other IgA-related responses exhibited differences near the nominal, unadjusted significance threshold (**Supplemental Figure 10**). When examining the relationships between these features among donors who recovered from mild, moderate, or severe disease, IgG-related features typically increased in magnitude uniformly alongside disease severity (**Figure 5B**). In contrast, the IgA-associated feature defined by nasal RBD-specific Abs binding to FcαR (**Figure 5C**) was lowest in subjects with either mild or severe disease, and elevated among a subset of those who recovered from moderate illness. Though differences between subjects experiencing moderate and mild disease or between those experiencing moderate and severe disease were not statistically significant (unpaired two-sided t-test with Welch’s correction), this apparent “goldilocks” profile raises the hypothesis that IgA responses may contribute to protection from less severe disease, but, like IgG responses, may not always be elicited either systemically or mucosally by mild disease.

### Functional Impact of Ab Isotype Depletion

**Figure 6:**
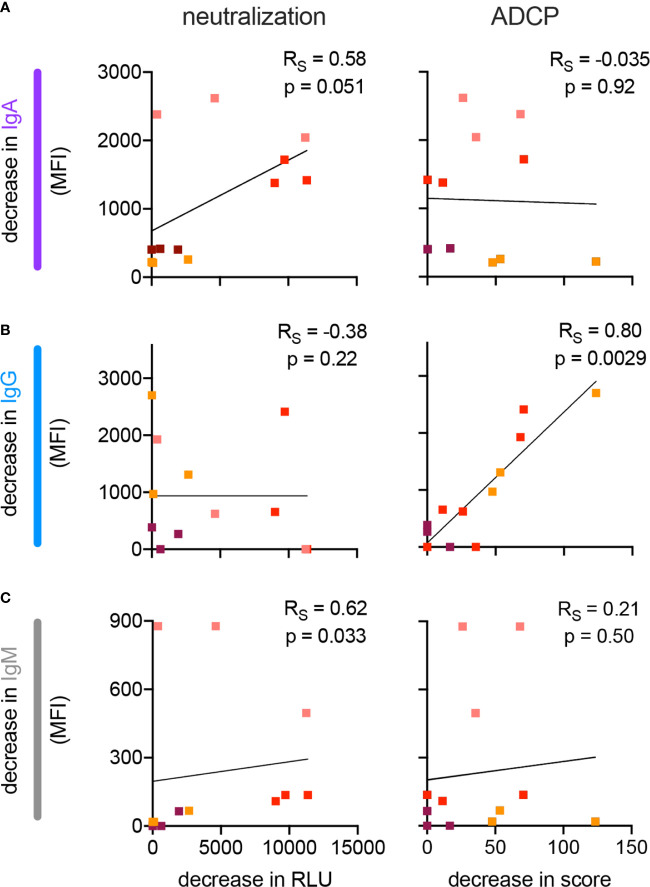
Impact of isotype depletion on antibody functions in nasal wash samples. **(A–C)** Scatterplots depicting the relationship between the extent of depletion of IgA **(A)**, IgG **(B)**, and IgM **(C)** and neutralization (left) and monocyte phagocytosis (right, ADCP) in nasal wash samples. Samples from four different individuals (indicated in color) were processed to deplete each of the three isotypes. Best fit line is illustrated. Spearman correlations (R_S_) and two-tailed significance values are indicated in inset.

Given the correlations between Ab isotypes and neutralization and other Ab-mediated effector functions, we sought to evaluate a mechanistic link by selective depletion of individual Ab isotypes from nasal wash samples. Samples from four subjects who had high neutralizing titers in both nasal wash and serum were selected for evaluation. Individual isotypes (IgG, IgA, IgM) were depleted *via* anti-isotype affinity resin and neutralization and phagocytic activity defined for mock treated and depleted samples (**Supplemental Figure 12**). The level of residual antibody in the depleted fractions was also quantified by multiplex assay. Because the extent of depletion of the intended isotype was variable and concomitant changes in other isotypes were observed, each depleted fraction was evaluated in comparison to a mock-depleted control to define the relationship between reduction of a given isotype and any subsequent decrease in neutralization or ADCP activity (**Figure 6**). Data points from each depletion were analyzed as though independent. Whereas depletion of IgG had no apparent impact on the neutralization potency of nasal wash samples, it directly correlated to decreases in phagocytosis (p = 0.0029). In contrast, depletion of IgA or IgM did not relate to changes in phagocytic activity, but was associated with reduced neutralization potency (p = 0.051 and p = 0.033, respectively). While these observations result from a limited number of individuals, they raise the possibility that mucosal IgA is a functional component of neutralization of SARS-CoV-2 in the upper respiratory tract, which is consistent with a wealth of prior data on CoV in human and animal studies (35–40).

## Discussion

A paradox emerging from studies of SARS-CoV-2 in naturally infected cohorts is that the highest Ab titers and most potent neutralizing Ab responses, known to associate with protection in animal models, have been observed in the serum of convalescent individuals who experienced severe infection (57, 59). Infected subjects with mild symptoms may not seroconvert (60–62). While information on systemic immune responses to SARS-CoV-2 continues to rapidly accrue, further questions remain about mucosal immune responses to the virus within the respiratory tract – the primary site of SARS-CoV-2 infection and replication (33) and where IgA is the dominant Ab isotype (63–65).

We sought to characterize the humoral immune response against SARS-CoV-2 with an emphasis on defining distinct features and functions associated with antibodies from the systemic and mucosal compartments. We examined the CoV-specific Ab response across a panel of SARS-CoV-2 antigens and four other endemic human CoVs. Intriguingly, though an effect of subject age and prior infection history cannot be excluded, Abs that bound to endemic CoV were elevated among SARS-CoV-2 convalescent donors when compared to naïve controls. While the early appearance of CoV-specific IgG in a subset of patients (66, 67) is suggestive of a recall response, data presented here cannot define whether these Abs are cross-reactive with other endemic CoV, or may represent a more general boosting phenomenon. Consistent with the hypothesis that boosted Abs are cross-reactive, SARS-CoV-2 S-reactive CD4^+^ T cells, a prerequisite for class-switched Ab responses, have been detected in the majority of COVID-19 patients and in 34% of uninfected individuals, supporting the existence of shared epitopes between S proteins of endemic CoV and SARS-CoV-2 (68–70). A similar phenomenon has been observed in immunofluorescence assays (11, 33). While many potential explanations exist, the observation that older individuals are most prone to severe COVID-19 illness, combined with the fact that the elderly have a decreased ability to generate *de novo* Abs (71–73), raises the possibility that pre-existing immunological memory from prior exposure to circulating CoVs (OC43, 229E, HKU1, and NL63) (74) resulting in the induction of poorly neutralizing but cross-reactive Abs may be associated with severe COVID-19 illness (75). To date, the results of other studies investigating this possibility have conflicted, with some concluding that recent endemic CoV infection could provide some benefit (76) while others suggest the absence of an impact (32). Thus, understanding the influence of pre-existing responses to endemic CoV remains an outstanding and important are of study.

Both multiplexed Ab profiling and functional assays showed induction of a generally robust humoral response against SARS-CoV-2 in the majority of subjects, but mild cases occasionally lacked serum (57, 61) or nasal responses. Indeed, the magnitude of the humoral response in serum appeared to be closely tied to the clinical severity of infection. Closer dissection of the humoral response revealed that it was primarily comprised of IgG1 and IgG3 – subclasses that readily promote effector function *via* their Fc domains and which were, along with FcγR-binding SARS-CoV-2-specific Abs, associated with diverse effector functions.

These observations suggest that SARS-CoV-2-specific Abs have the potential to contribute to protection against COVID-19 through the involvement of cells of the innate immune system and the complement system, and not solely by neutralization. While much has been made of the potential for Ab responses to promote infection or inflammation *via* interactions with FcRs or *via* other mechanisms (77–79), it remains unclear whether the elevated IgG response magnitude is a cause or effect of increased disease severity (80). Limitations to functional characterization reported here include use of surrogate endpoints of anti-viral activities, such as the substitution of FcγR3a activation and complement C3b deposition as alternatives to assessing infected cell death or viral lysis, and the use of pseudovirus in neutralization tests and recombinant S proteins in effector function assays. However, assays simplified in these ways have served as correlates of vaccine efficacy in multiple human and animal model studies (81–86) and represent approaches readily available for deployment in the global effort to understand responses to SARS-CoV-2 infection and define protective immunity.

Independent of where antibodies fall on a protective-pathogenic spectrum, the characteristics of the responses observed in serum and nasal samples tended to be highly distinct. Not only did the Ab profiles from individual subjects tend to favor either IgG or IgA, these isotypes showed variable relationships to neutralization activity depending on the site. SARS-CoV-2-specific IgG in serum was associated with neutralization, consistent with prior work demonstrating that neutralizing serum antibodies targeting SARS-CoV-1 and SARS-CoV-2 are highly correlated with IgG response magnitude (57, 87). In contrast, those subjects who mounted a relatively IgA-biased nasal response exhibited elevated nasal wash neutralization activity, suggesting that the mechanistic contribution of mucosal IgA to neutralization of virus at the portal of entry could be substantial. To this end, while confounded by concomitant effects on unintended isotypes, depletion experiments support the mechanistic relevance of IgG responses to mucosal Ab-mediated phagocytosis, and IgA and IgM to mucosal Ab-mediated neutralization. The observation that those subjects whose nasal specimens had the greatest neutralization potency also tended to report experiencing only mild or moderate symptoms suggests that virus neutralization by mucosal IgA could be relevant to disease outcomes. It is important to note however, that the available cohort was not large enough to adequately power a robust examination of this trend, and further studies will be needed to support this association. Reduced SARS-CoV-2-specific humoral responses among subjects with mild disease also confound the ability to identify potential correlates of reduced severity of disease among these convalescent donors. Several individuals appeared not to “naso” convert, and one mild case failed to seroconvert according to clinical testing. Further, because of the many correlations observed between Ab types and activities in different anatomical sites, additional depletion and other follow up studies will be needed to further define the mechanistic relevance of feature-function correlations and their potential to make causal contributions to modifying disease severity. Though IgA responses may show poorer durability than IgG (88, 89), taken together with prior studies of other CoV in humans and animals (35–40), these data raise the possibility that levels of SARS-CoV-2-specific mucosal IgA could serve as a useful immune correlate for mitigated disease severity, protection from infection, and reduced likelihood of transmission.

These data have important implications for our understanding of the protection afforded by vaccination or prior infection. When considering vaccine development, an ideal candidate would not only protect the recipient from disease but would also prevent them from serving as an asymptomatic vector, as can be the case in other vaccine-treatable diseases such as polio and pertussis (90–92). Polio is a particularly informative model in this respect as the mucosally-administered and actively replicating form of the vaccine is capable of providing sterilizing immunity—at the expense of a risk of reversion to virulence—while the systemically administered inactivated vaccine fails to induce mucosal immunity and thus serves primarily to protect the recipient from neurological sequelae (58). Our current observation that natural infection elicits alternatively IgG or IgA-biased responses, with IgG associated with serum neutralization potency but severe disease and IgA associated with nasal neutralization activity and mild to moderate disease, suggests that such a dichotomy could exist for COVID-19 as well. While correlates of protection against SARS-CoV-2 in humans have yet to be defined, lessons from related CoV in animals and humans are consistent with the results of this small natural infection history study; mucosal IgA is likely of substantial importance.

## Data Availability Statement

The original contributions presented in the study are included in the article/**Supplementary Material**. Further inquiries can be directed to the corresponding authors.

## Ethics Statement

The studies involving human participants were reviewed and approved by Dartmouth College and Dartmouth-Hitchcock Medical Center Committee for the Protection of Human Subjects. The patients/participants provided their written informed consent to participate in this study.

## Author Contributions

Conceptualization: PW and MA. Investigation: SB, AC, HN, WW-A, and RC. Writing—original draft: SB, AC, HN, JL, MA. Writing—review and editing: all authors. Data analysis and curation: SB, AC, HN, SX, JW, CB, DM, RC. Supervision: PW and MA. Project Administration: JW, WW-A. Funding acquisition: SB, PW, and MA. All authors contributed to the article and approved the submitted version.

## Funding

This work was supported by NIH NCI supplement to 2 P30 CA 023108-41, the BioMT Molecular Tools Core supported by NIGMS COBRE award P20-GM113132, Bill and Melinda Gates’ Foundation OPP1104756, and by DHMC for providing support for collection of initial cohort samples. SB is supported by NIH NIAID 2T32AI007363.

## Conflict of Interest

The authors declare that the research was conducted in the absence of any commercial or financial relationships that could be construed as a potential conflict of interest.
