# Supplementary material for: Distinct Features and Functions of Systemic and Mucosal Humoral Immunity Among SARS-CoV-2 Convalescent Individuals
Source: Front Immunol. 2021 Jan 28;11:618685. doi: 10.3389/fimmu.2020.618685 (PMC7876222; doi:10.3389/fimmu.2020.618685)
Supplement: Supplementary file 1 [file DataSheet_1.pdf]

Supplementary Materials

**Supplementary Figures**	
Supplementary Figure 1	Serum IgG isotype and subclass responses across CoV and control antigen types.
Supplementary Figure 2	Serum IgA, D, and M isotype and subclass responses across CoV and control antigen types.
Supplementary Figure 3	Serum FcγR binding responses across antibodies specific to CoV and control antigen types.
Supplementary Figure 4	Nasal IgG isotype and subclass responses across CoV and control antigen types.
Supplementary Figure 5	Nasal IgA, D, and M isotype and subclass responses across CoV and control antigen types.
Supplementary Figure 6	Nasal FcγR binding responses across antibodies specific to CoV and control antigen types.
Supplementary Figure 7	Stool IgA and IgG responses across CoV and control antigen types
Supplementary Figure 8	Differences in Ab responses observed between naïve and convalescent donors
Supplementary Figure 9	Correlations between Ab features within serum and nasal wash samples
Supplementary Figure 10	Differences in Ab responses among convalescent donors by subject characteristics
Supplementary Figure 11	Heatmap of serum Ab features that differ between donors who experienced severe versus non-severe disease
Supplementary Figure 12	Impact of isotype depletion on nasal wash neutralization and phagocytosis
**Supplementary Tables**	
Supplementary Table 1	Demographic data for convalescent and naive donors
Supplementary Table 2	Fc Array Reagents
Supplementary Table 3	CoV Antigen Sequences

Supplementary Table 4	Sample Dilutions in the Fc Array Assay

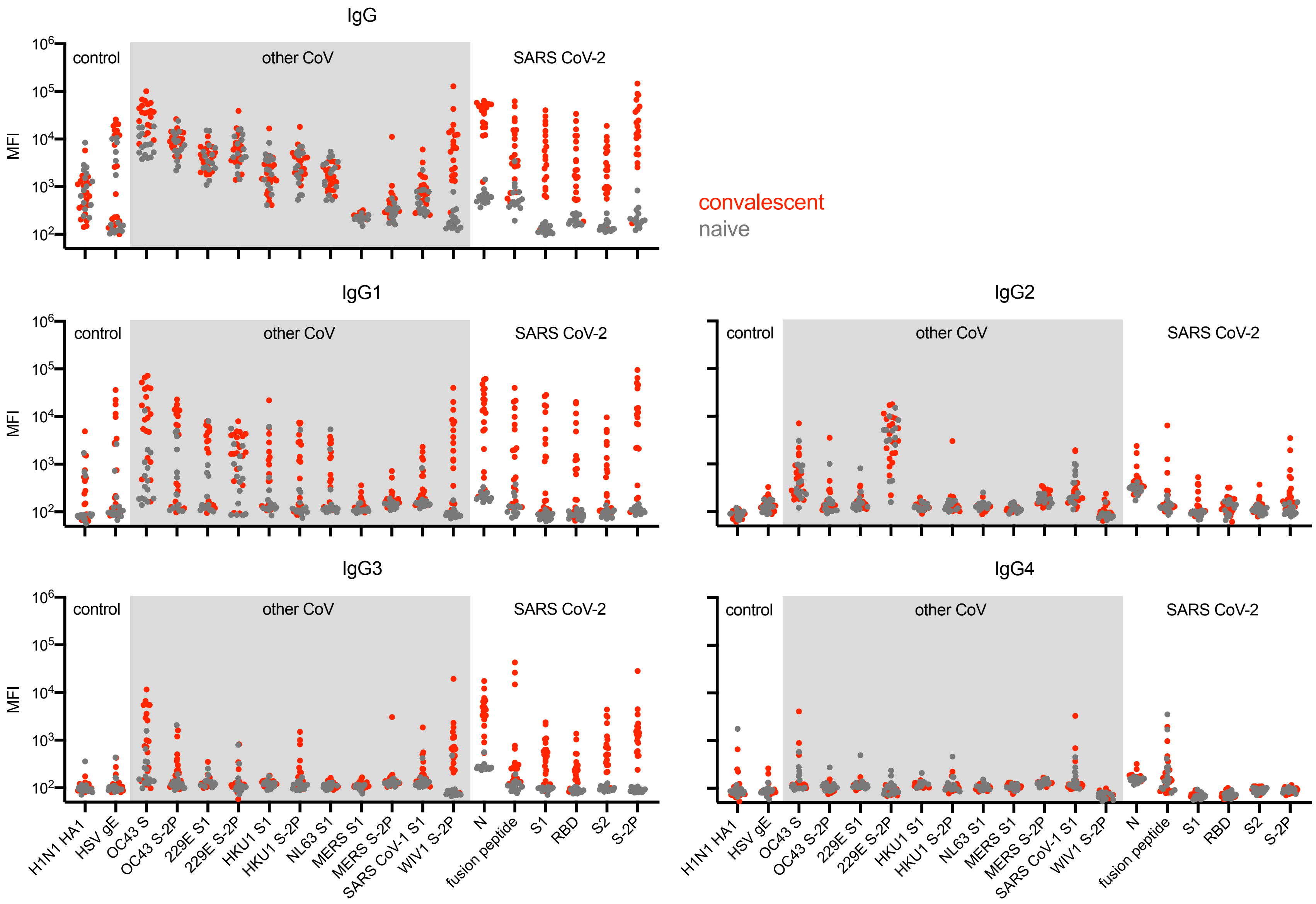

Supplementary Figure 1. Serum IgG isotype and subclass responses across CoV and control antigen types.

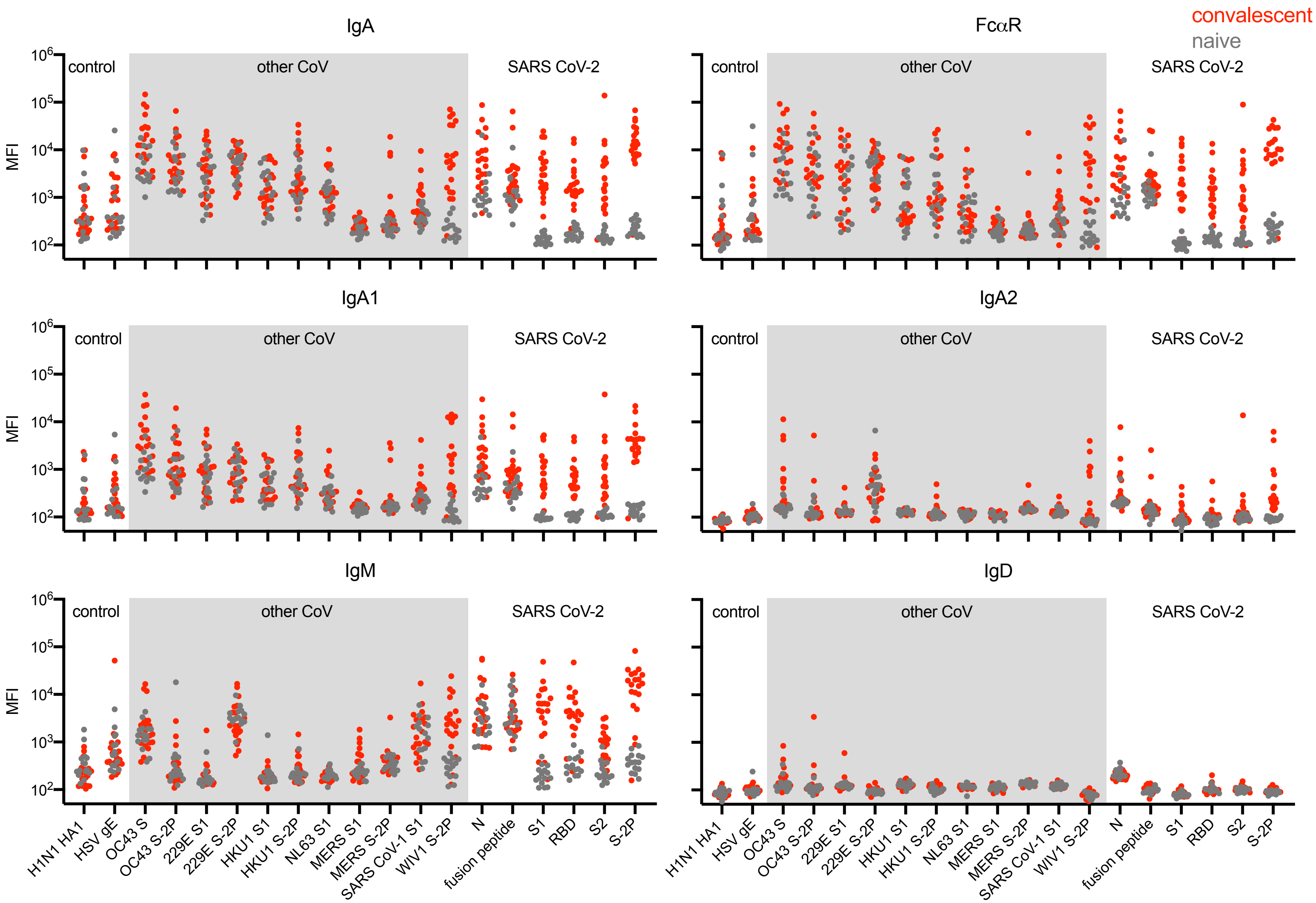

Supplementary Figure 2. Serum IgA, D, and M isotype and subclass responses across CoV and control antigen types.

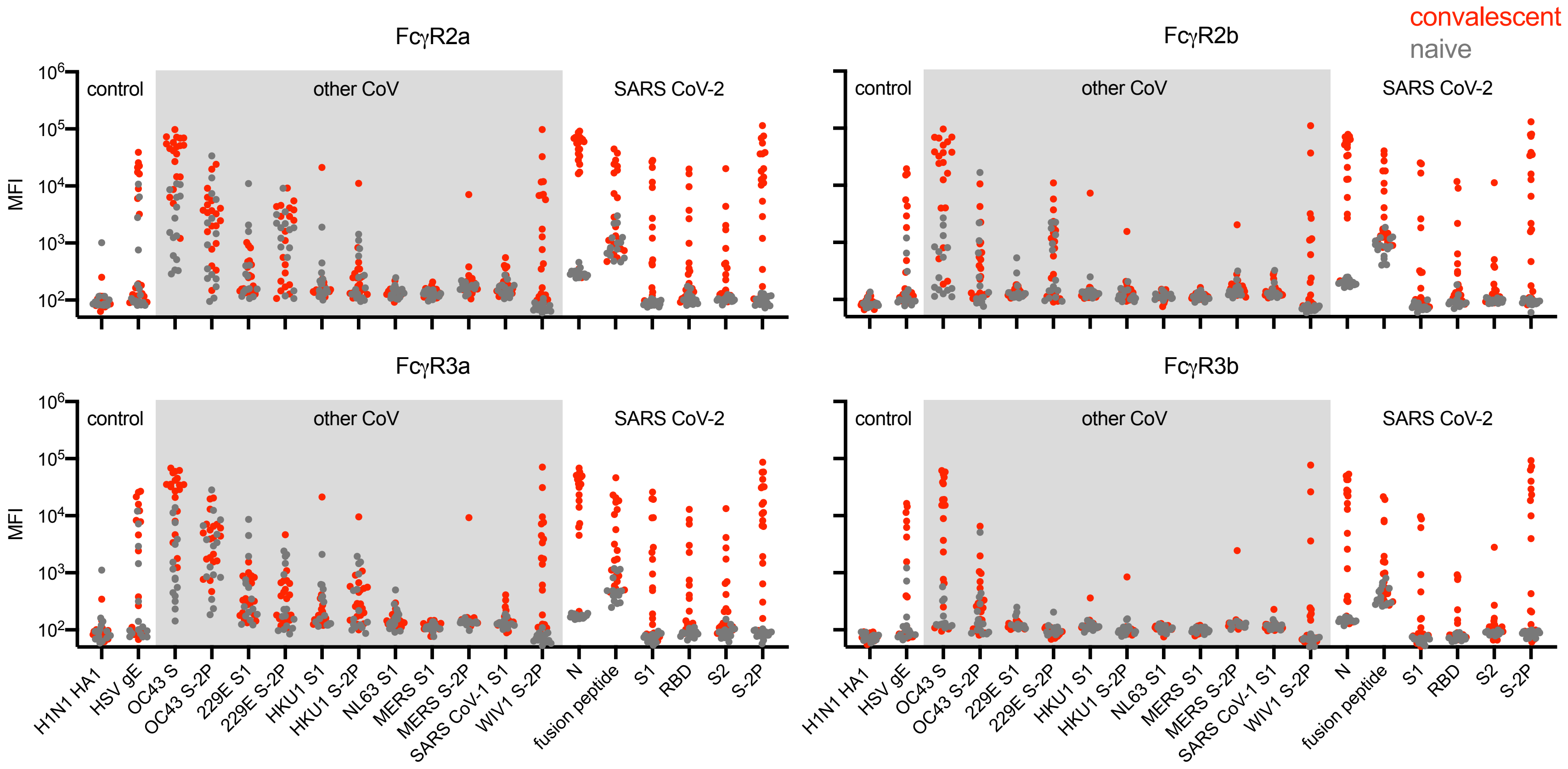

Supplementary Figure 3. Serum FcγR binding responses across antibodies specific to CoV and control antigen types.

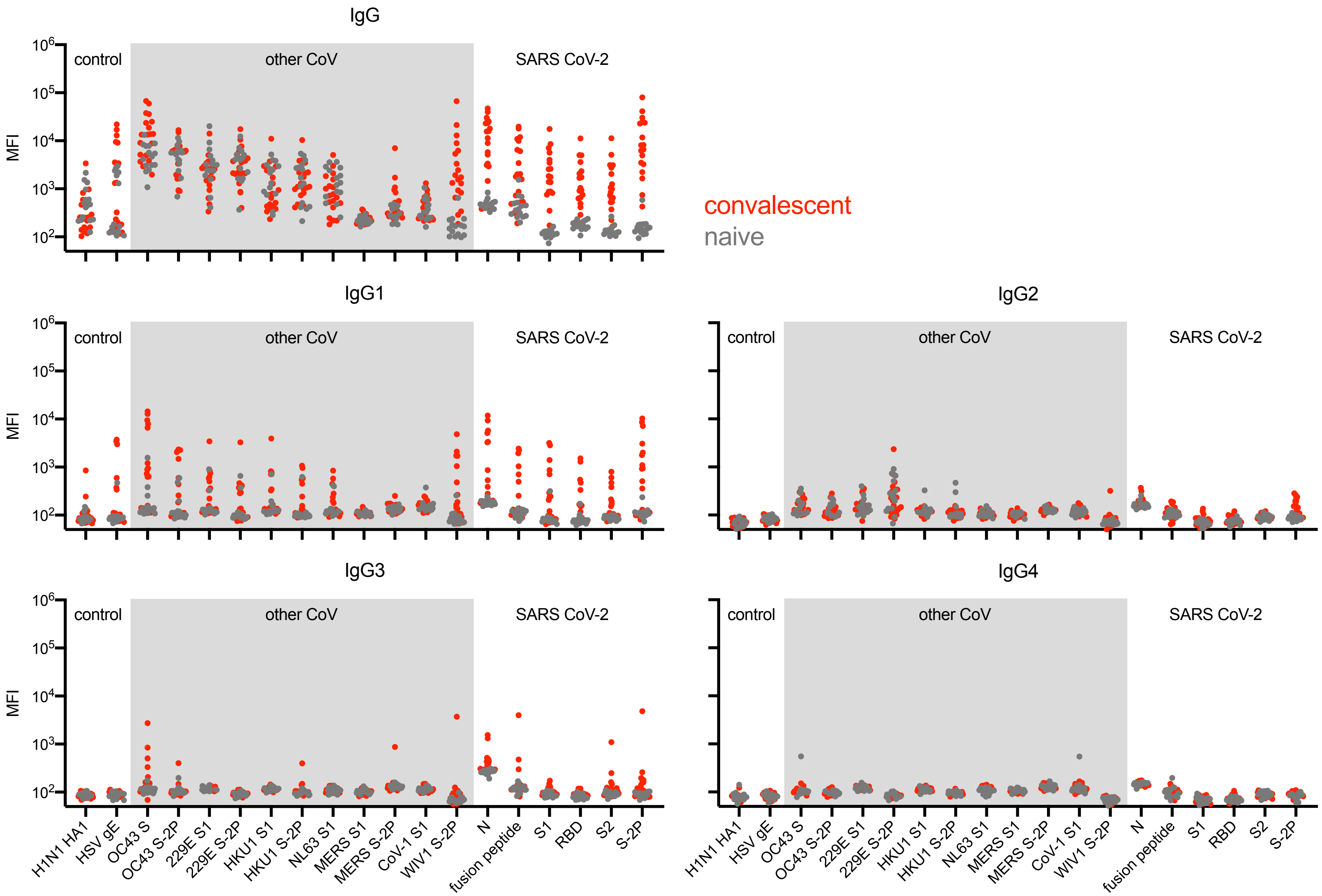

Supplementary Figure 4. Nasal IgG isotype and subclass responses across CoV and control antigen types.

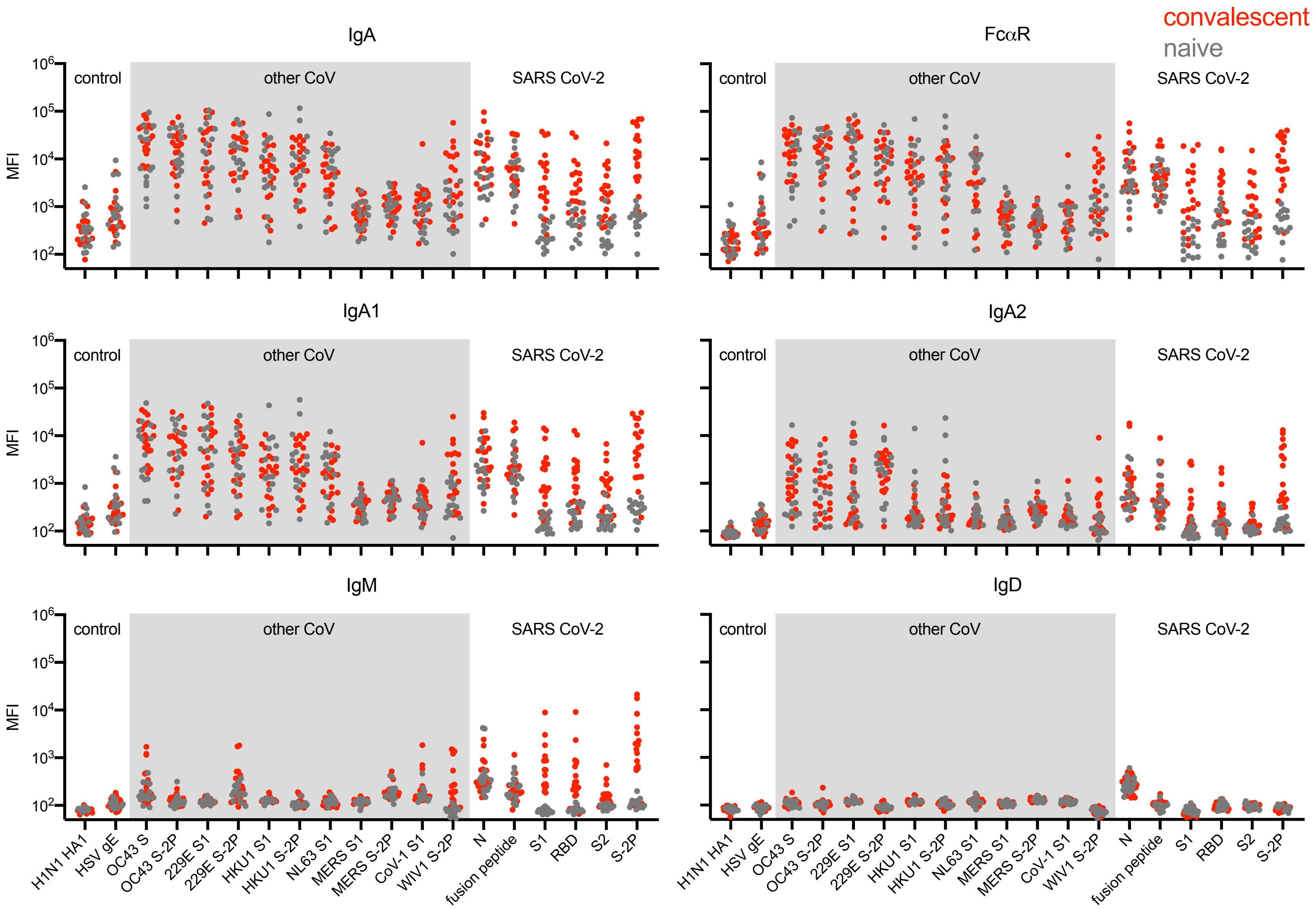

Supplementary Figure 5. Nasal IgA, D, and M isotype and subclass responses across CoV and control antigen types.

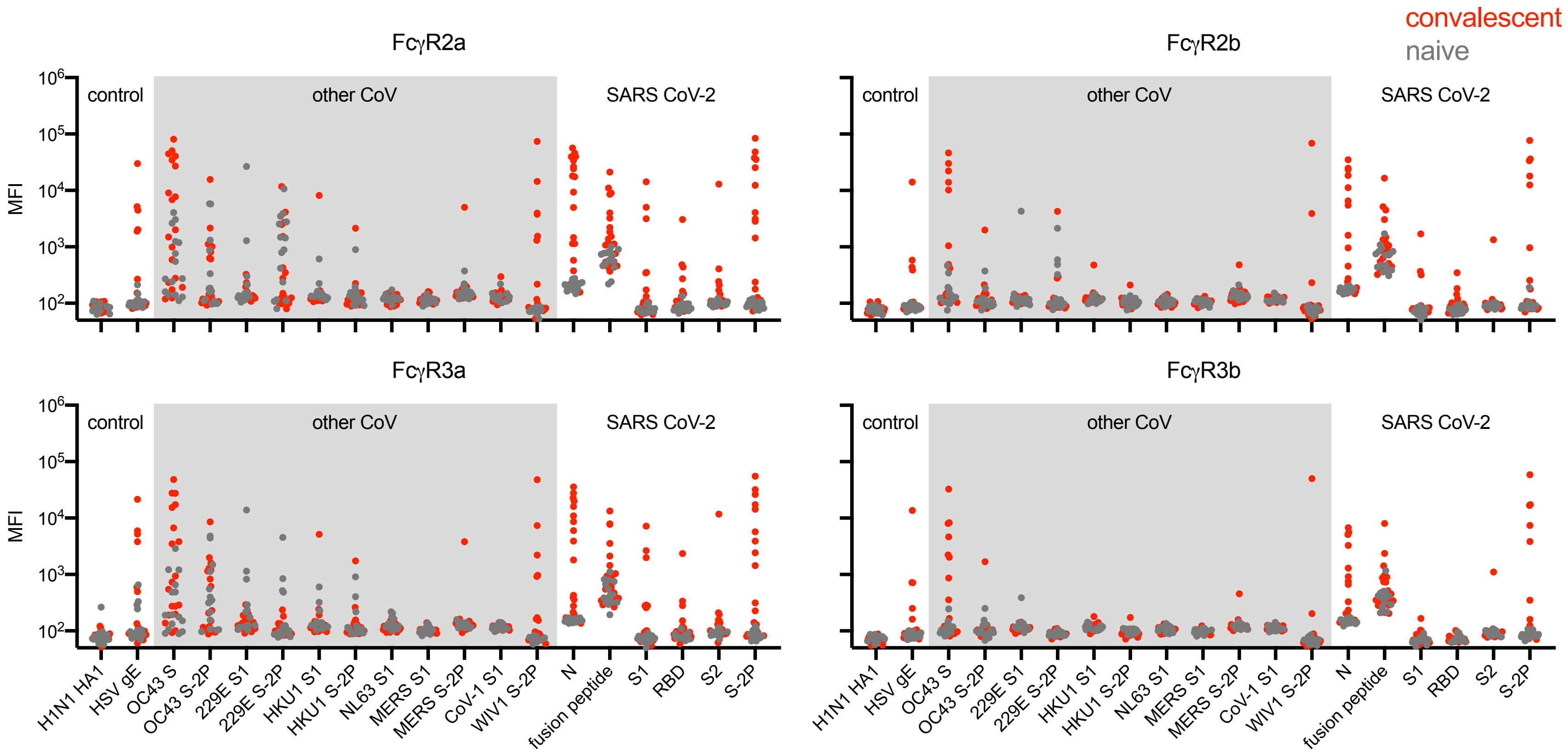

Supplementary Figure 6. Nasal FcγR binding responses across antibodies specific to CoV and control antigen types.

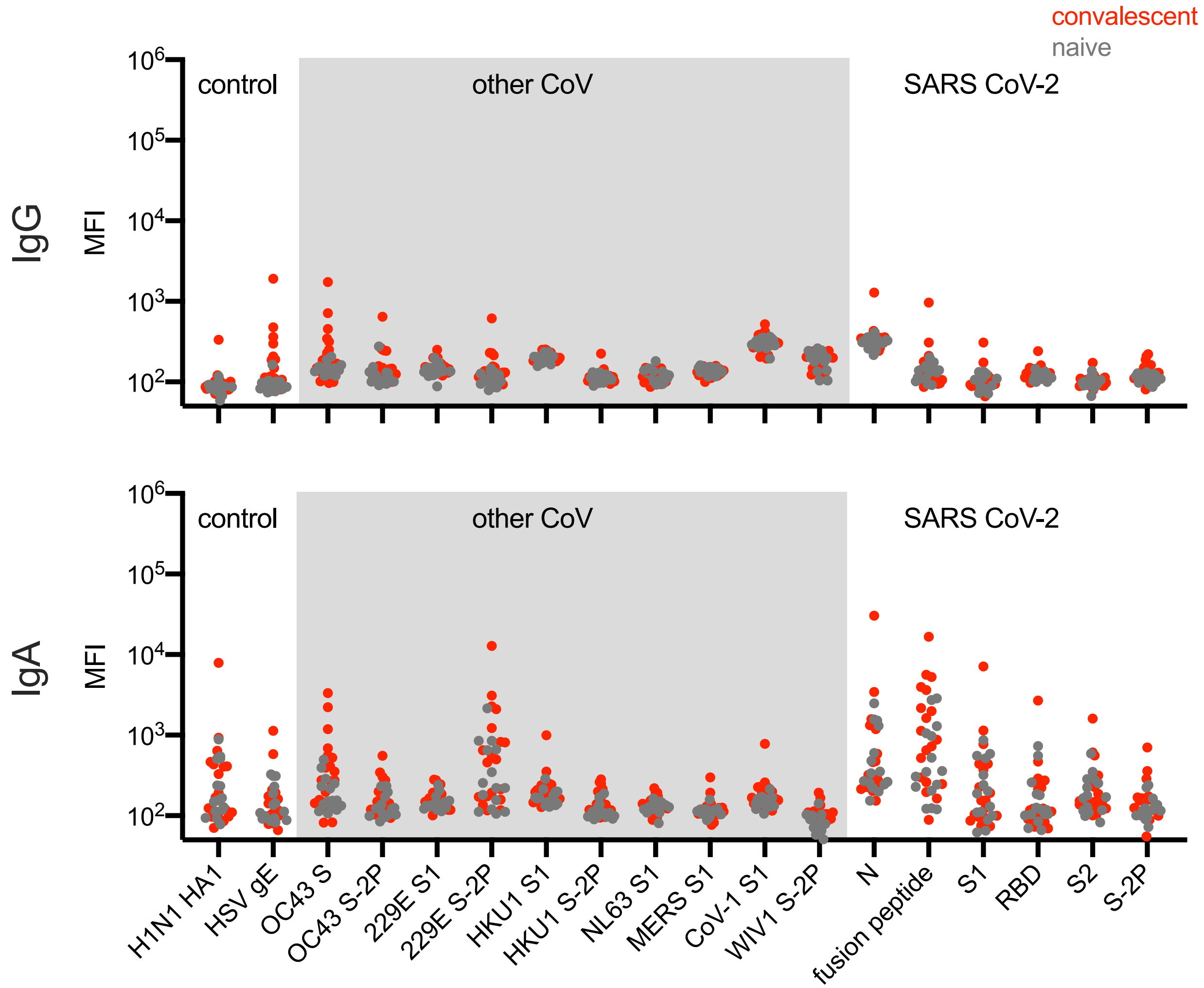

Supplementary Figure 7. Stool IgG and IgA responses across CoV and control antigen types.

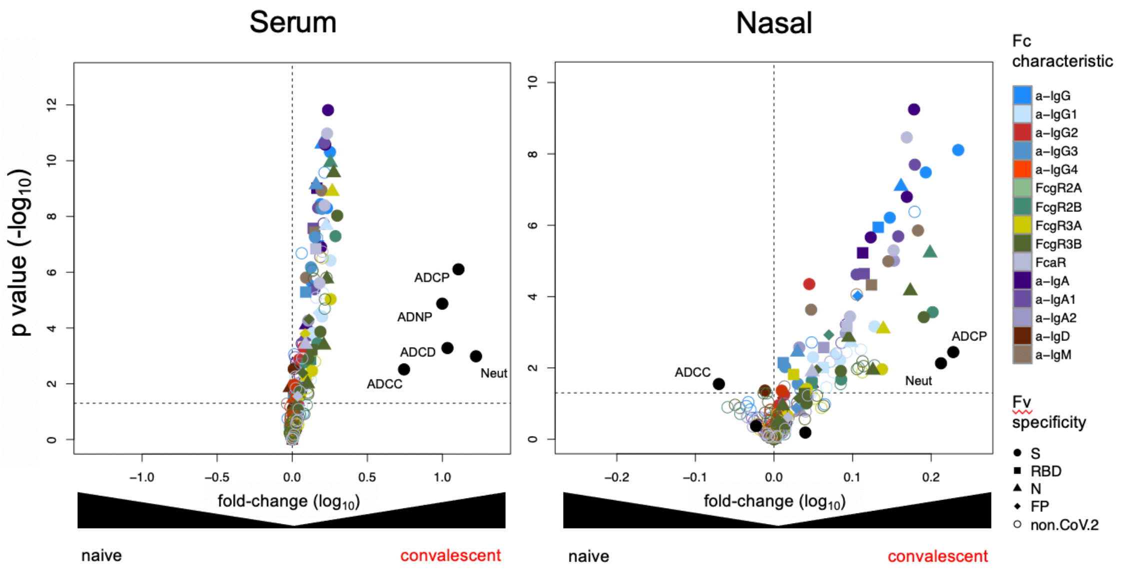

Supplementary Figure 8. Differences in Ab responses observed between naïve and convalescent donors. Volcano plots depicting fold-change and unadjusted statistical significance (unpaired two-tailed t test with Welch's correction) of biophysical and functional response features in serum (left) and nasal wash (right) samples by infection status. Functional assay data is labeled by name and log-transformed Fc array data is depicted with antigen specificity indicated by shape and Fc characteristic indicated by color. Dotted horizontal line indicates p = 0.05.

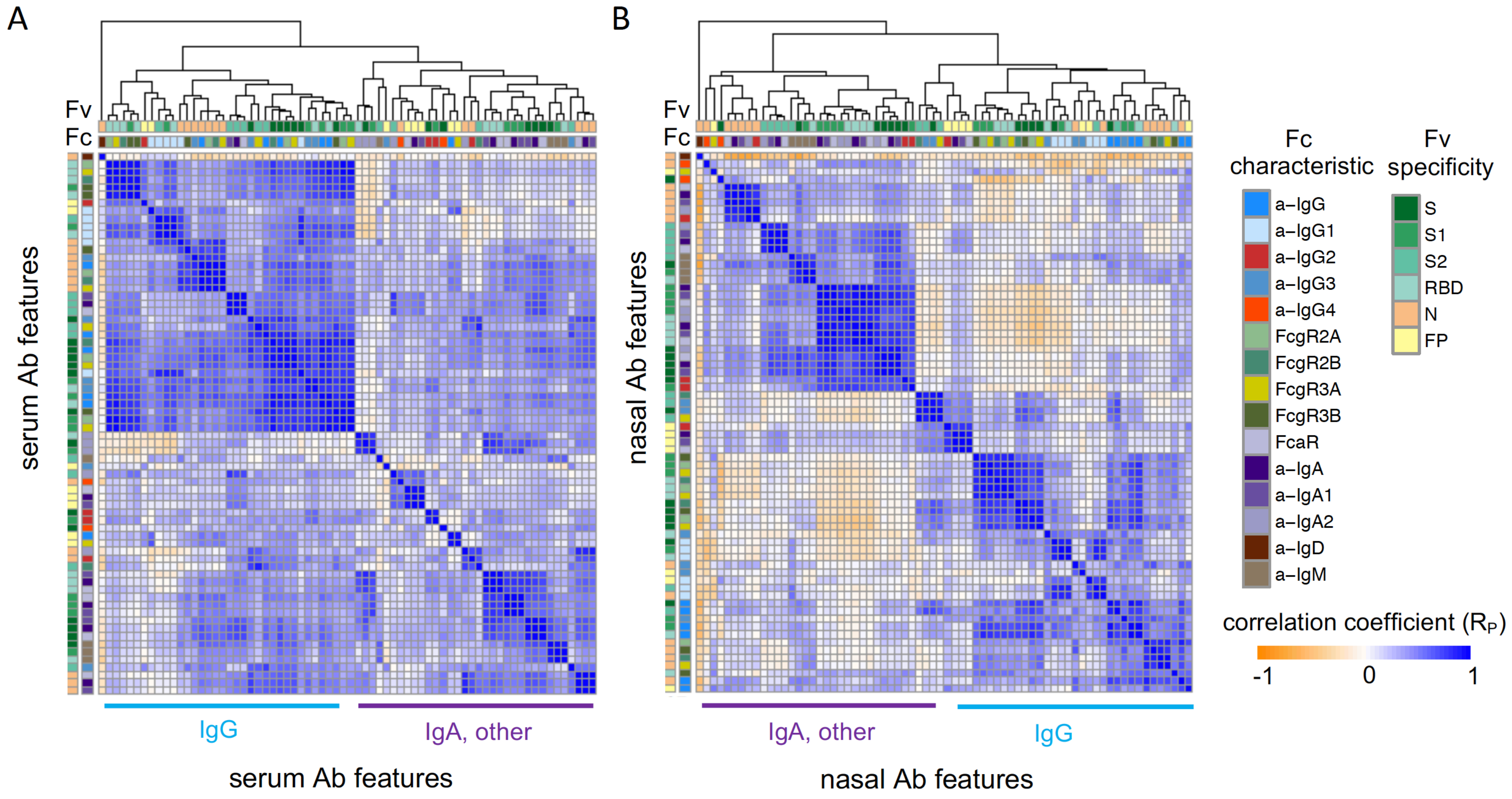

Supplementary Figure 9. Correlations between Ab features within serum and nasal wash samples. A-B. Correlation matrices of relationships between Ab response features measured in serum (**A**) and nasal wash (**B**) samples from convalescent donors. Antigen specificity (Fv) and Fc characteristics (Fc) are indicated in color bars. Filtered CoV-2-specific Ab features are hierarchically clustered. Pearson correlation coefficients (RP) are shown. Dominant isotype(s) of main branches of dendrograms are indicated.

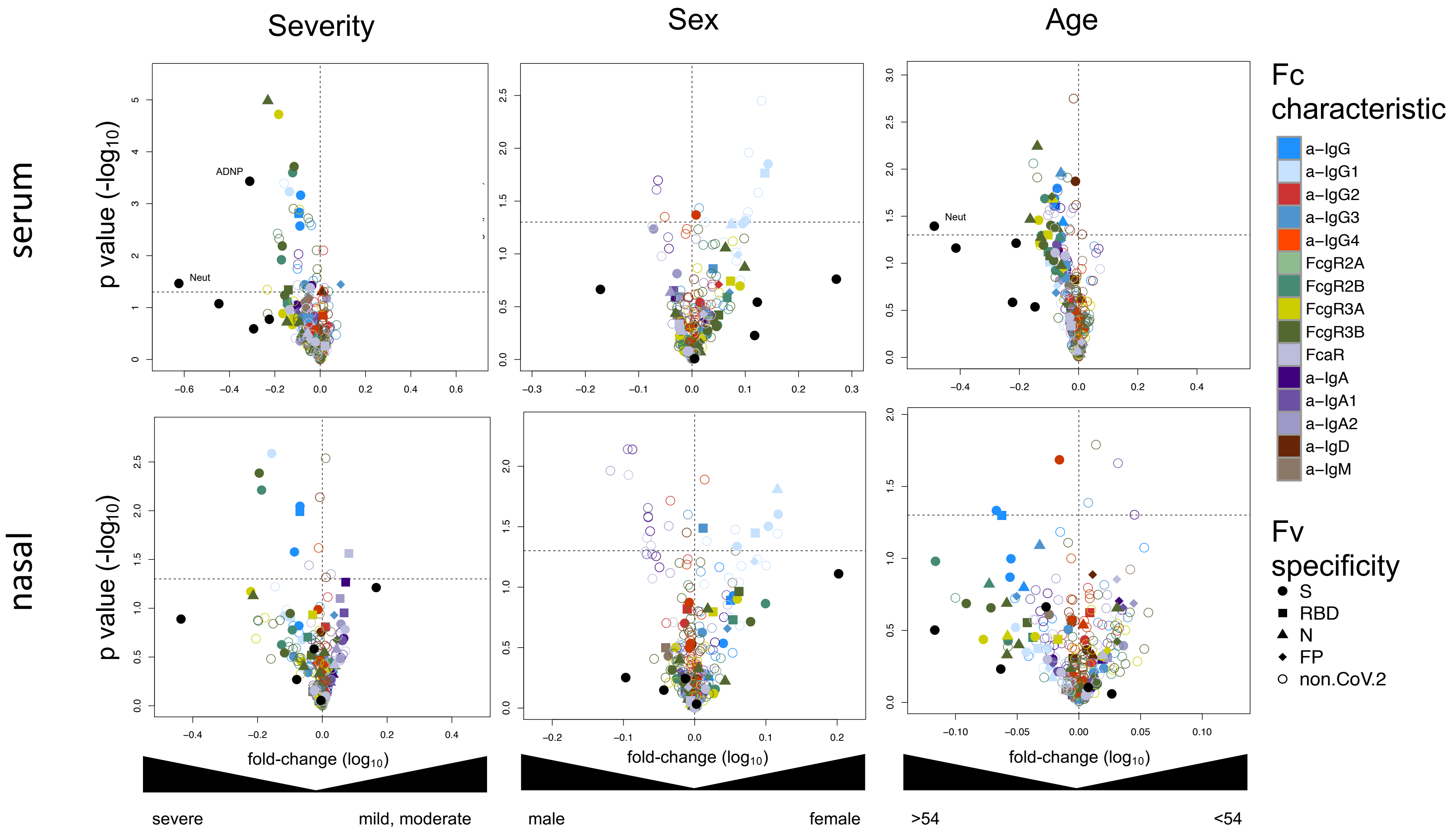

Supplementary Figure 10. Differences in Ab responses among convalescent donors by subject characteristics. Volcano plots depicting fold-change and unadjusted statistical significance (unpaired two-tailed t test with Welch's correction) of biophysical and functional response features in serum (top) and nasal wash (bottom) samples by disease severity (left), sex (center), and age (right). Functional assay data is labeled by a black circle with the assay name added if $p < 0.05$, and Fc array data is depicted with antigen specificity indicated by shape and Fc characteristic indicated by color. Dotted horizontal line indicates $p = 0.05$.

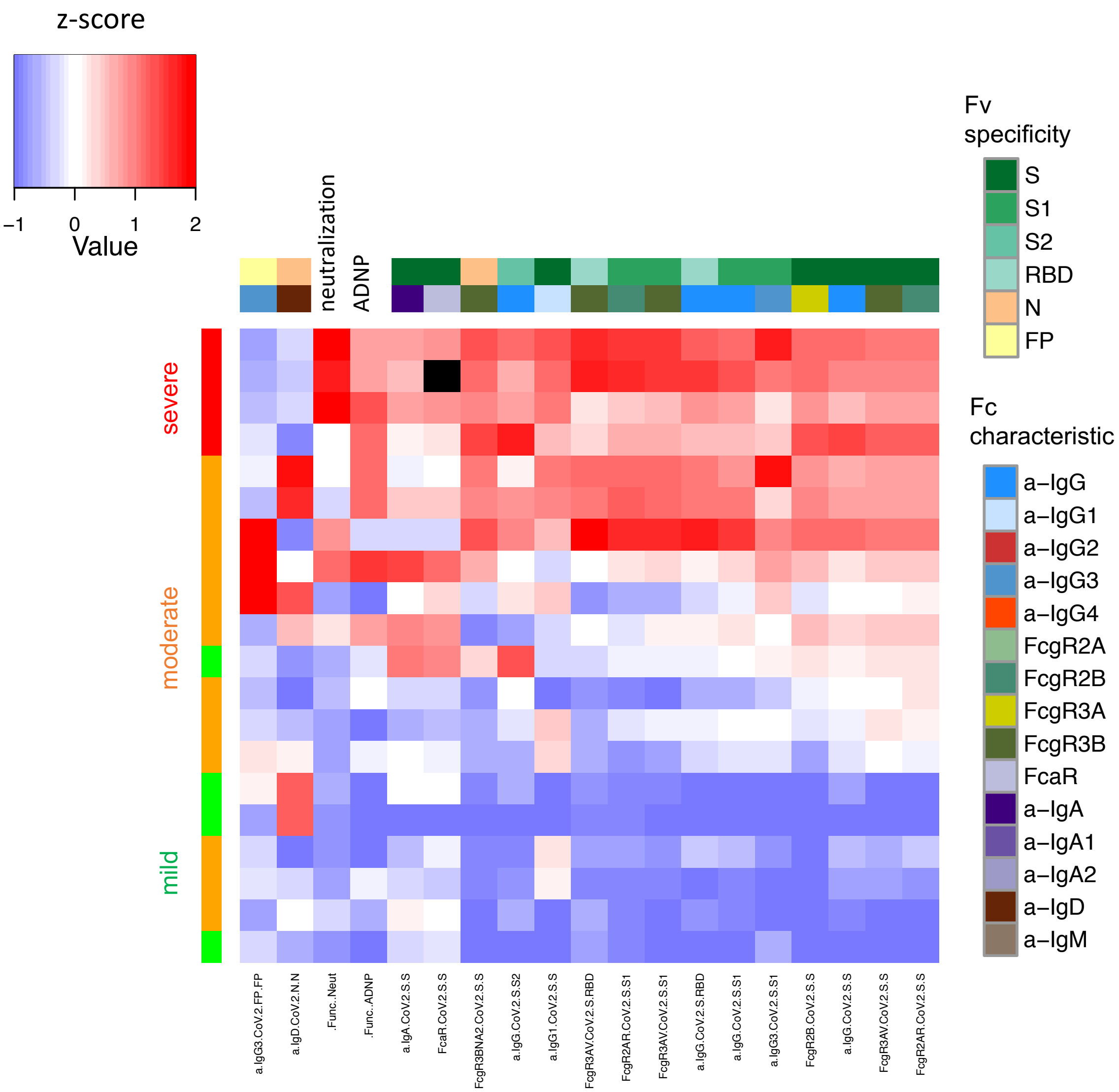

Supplementary Figure 11. Heatmap of serum Ab features that differ between donors who experienced severe versus non-severe disease. Features and subjects are hierarchically-clustered with Fv specificity (viral antigen) and Fc characteristic (detection) indicated in x-axis color bar. Subjects are stratified by infection or disease severity status. Responses are centered and scaled per feature and the scale range truncated at +2/-1 SD. Black cell indicates missing data.

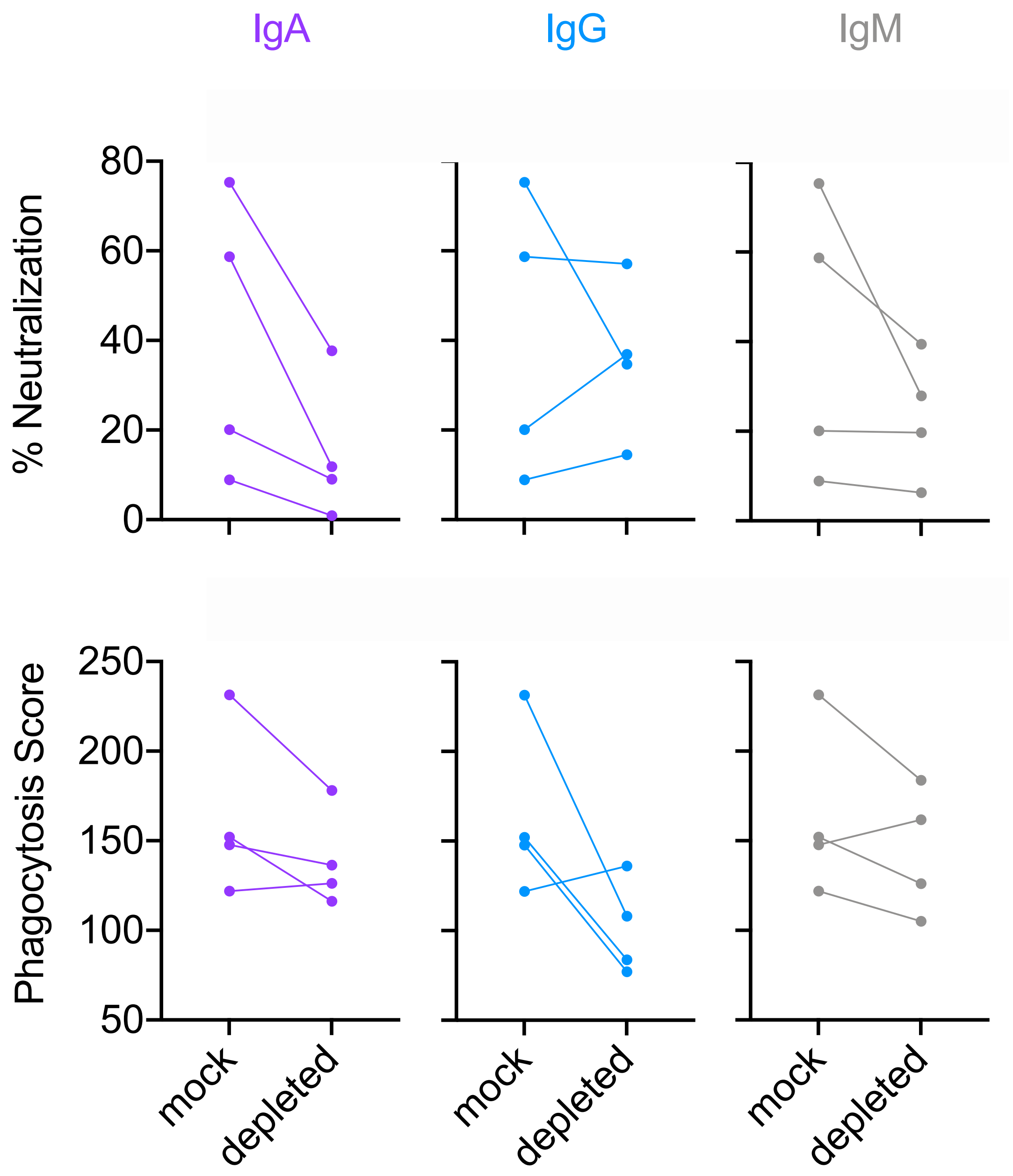

Supplementary Figure 12. **Impact of isotype depletion on nasal wash neutralization and phagocytosis**. Neutralization (top) and phagocytosis (bottom) activity observed in select nasal wash samples with (depleted) or without (mock) IgA (left), IgG (center), or IgM (right) depletion.

Supplementary Table 1. Demographic data for convalescent and naive donors

Characteristic	Convalescent			Naïve
	**Severe**	**Moderate**	**Mild**	
	n=4	n=12	n=4	n=15
Median age (IQR), years	66.5 (49-77)	54 (18-74)	26 (26-59)	34 (28-52)
Sex				
Female	2 (50%)	7 (58.33%)	1 (25%)	8 (53.3%)
Male	2 (50%)	5 (41.66%)	3 (75%)	7 (46.7%)
Median days since symptom onset (IQR)	40.5 (29-56)	38 (30-67)	31 (19-42)	Not applicable

Supplementary Table 2. Antigen and Fc Detection Reagents

Antigen	Source	Fc Detection	Source
H1N1 HA1	Immune Technology IT-003-00110p	a- IgG	Southern Biotech 1030-09
HSV gE	Immune Technology IT-005-005p	a-IgG1	Southern Biotech 9054-09
HCoV OC43 S	Sino Biological 40607-V08B	a-IgG2	Southern Biotech 9070-09
OC43 S-2P	Expressed in HEK 293F	a-IgG3	Southern Biotech 9210-09
229E S1	Sino Biological 40605-V08H	a-IgG4	Southern Biotech 9200-09
229E S-2P	Expressed in Expi 293	a-IgA	Southern Biotech 2050-09
HKU1 S1	Sino Biological 40606-V08H	a-IgA1	Southern Biotech 9130-09
HKU S-2P	Expressed in Expi 293	a-IgA2	Southern Biotech 9140-09
NL63 S1	Sino Biological 40604-V08H	a-IgM	Southern Biotech 9020-09
MERS S1	Sino Biological 40069-V08H-50	a-IgD	Southern Biotech 9030-09
MERS S-2P	Expressed in Expi 293	FcαR	Duke Protein Production Facility
SARS CoV-1 S1	Sino Biological 40150-V08B1	FcγR2a	Boesch, et. al, 2014[1]
WIV1 S-2P	Expressed in Expi 293	FcγR2b	Boesch, et. al, 2014[1]
SARS CoV-2 N	Immune Technology IT-002-033Ep	FcγR3a	Boesch, et. al, 2014[1]
SARS CoV-2 fusion peptide	New England Peptide	FcγR3b	Boesch, et. al, 2014[1]
SARS CoV-2 S1 (for Fc Array)	ACROBiosystems S1N-C52H3-100ug		
SARS CoV-2 S1 (for ADCD)	Sino Biological 40150-V08B1-20		
SARS CoV-2 RBD	BEI Resources NR-52366		
SARS CoV-2 S2	Immune Technology IT-002-034p		
SARS CoV-2 S-2P	Expressed in Expi 293		

[1]Boesch AW, Brown EP, Cheng HD, Ofori MO, Normandin E, Nigrovic PA, et al. Highly parallel characterization of IgG Fc binding interactions. MAbs. 2014;6(4):915-27.

Supplementary Table 3. CoV Antigen Sequences

Antigen	Sequence (5’ → 3’)
SARS-CoV-2 (S-2P)	ATGTTCGTGTTCCTGGTGCTCCTGCCTCTGGTGAGCAGCCAGTGCGTGAACCTG<br>ACCACCCGAACCCAGCTCCCACCAGCCTACACCAACAGCTTTACACGGGGCGTG<br>TACTACCCTGACAAGGTGTTCAGATCTAGCGTCCTGCACAGCACTCAGGACCTCT<br>TCCTGCCGTTCTTCAGCAACGTGACATGGTTCCACGCCATCCACGTGAGCGGCA<br>CAAACGGAACCAAGCGGTTTGATAACCCCGTCCTGCCATTCAATGATGGAGTTTA<br>CTTCGCCAGTACCGAGAAGAGTAACATCATCCGGGGCTGGATCTTCGGCACCAC<br>CCTGGATAGCAAAACACAGAGCCTCCTGATCGTGAACAATGCCACGAACGTCGT<br>GATCAAGGTGTGCGAGTTCCAGTTTTGCAATGATCCTTTCCTGGGTGTGTACTAC<br>CACAAGAACAACAAGAGCTGGATGGAAAGCGAGTTCAGAGTCTACAGCAGCGCC<br>AACAACTGCACATTCGAGTACGTCTCTCAGCCTTTTCTGATGGACCTTGAGGGGA<br>AACAAGGCAACTTCAAGAACCTGAGAGAATTCGTGTTCAAGAACATCGACGGCTA<br>CTTCAAAATCTACTCCAAGCACACACCCATCAACCTGGTCCGGGACCTCCCTCAG<br>GGCTTCAGCGCCCTGGAACCCCTGGTCGACCTGCCCATAGGCATCAACATAACG<br>CGGTTCCAAACCCTGCTGGCCCTGCATAGATCCTACCTGACTCCTGGCGACAGC<br>AGCAGCGGATGGACCGCCGGAGCTGCAGCCTACTATGTGGGCTACCTGCAACC<br>TAGAACCTTCCTGCTGAAGTACAACGAGAACGGCACAATCACAGACGCCGTCGA<br>CTGCGCCCTGGACCCTCTCTCTGAGACAAAGTGCACCCTGAAGTCCTTCACCGT<br>GGAAAAGGGCATCTACCAGACCAGCAACTTCCGGGTGCAGCCTACAGAGAGCAT<br>CGTGCGATTTCCAAACATTACCAACCTCTGCCCCTTCGGCGAGGTGTTTAACGCC<br>ACAAGATTTGCCTCCGTTTACGCCTGGAATAGAAAGAGAATCAGCAATTGTGTGG<br>CCGACTACTCCGTGCTGTATAACAGCGCCTCTTTCAGCACCTTCAAGTGCTACGG<br>CGTTTCCCCAACAAAGCTGAATGACCTGTGCTTCACCAACGTGTACGCCGACTCC<br>TTCGTAATTAGAGGCGATGAGGTGCGGCAGATCGCACCAGGCCAGACCGGTAA<br>GATCGCTGACTACAACTATAAGCTGCCTGATGATTTTACAGGCTGCGTGATCGCC<br>TGGAACTCTAACAACCTGGATAGCAAGGTGGGCGGCAACTACAACTACCTGTAC<br>CGGCTGTTTCGCAAGTCTAACCTGAAACCTTTCGAGAGAGACATCTCCACAGAGA<br>TCTACCAGGCCGGTTCTACACCTTGTAACGGGGTGGAAGGCTTCAACTGTTACTT<br>CCCTCTGCAAAGCTACGGCTTCCAGCCTACCAATGGAGTCGGCTACCAGCCATA<br>CCGGGTGGTCGTGCTGTCCTTCGAGTTACTCCACGCCCCCGCCACCGTCTGCG<br>GTCCTAAGAAGTCCACCAATCTGGTTAAGAACAAATGCGTGAACTTCAACTTCAA<br>CGGCCTGACCGGGACCGGCGTGCTGACCGAAAGCAACAAAAAGTTCCTCCCCTT<br>CCAGCAGTTCGGCCGTGATATCGCTGACACCACAGATGCCGTCAGAGATCCACA<br>GACCCTGGAAATCCTGGATATTACACCCTGCTCCTTCGGAGGAGTTTCTGTGATC<br>ACCCCCGGGACCAATACCAGCAACCAGGTGGCTGTGCTGTACCAAGATGTTAAC<br>TGCACCGAGGTTCCTGTGGCCATCCACGCCGATCAGCTGACACCTACTTGGAGA<br>GTGTACTCCACTGGCTCCAATGTGTTCCAGACCAGGGCCGGATGTCTGATCGGC<br>GCCGAGCACGTGAATAACAGTTACGAGTGCGACATCCCTATCGGCGCCGGCATC<br>TGTGCCAGCTACCAGACCCAGACAAACAGCCCTGGGTCTGCTTCCTCTGTAGCT<br>AGCCAGAGCATCATCGCCTACACCATGAGCCTGGGCGCAGAGAACAGCGTGGC<br>CTATTCCAACAACTCTATCGCCATTCCCACCAACTTTACAATTAGCGTCACAACAG<br>AGATCCTGCCCGTGAGCATGACCAAGACCAGCGTGGACTGTACAATGTACATCT<br>GTGGCGACAGCACTGAATGCAGCAACCTGCTGCTGCAATACGGCTCCTTTTGCA<br>CCCAACTGAACCGGGCGCTGACCGGAATCGCCGTGGAACAGGACAAAAATACC<br>CAGGAGGTGTTCGCCCAAGTGAAGCAGATCTACAAGACCCCACCTATCAAGGAC<br>TTCGGCGGCTTTAACTTTAGCCAGATTCTCCCTGATCCTTCTAAGCCTAGCAAGC<br>GGAGCTTTATCGAGGATCTGCTGTTCAACAAGGTCACCCTGGCCGATGCCGGCT<br>TTATCAAACAGTATGGCGATTGCCTGGGCGACATAGCCGCCAGAGATCTGATCT<br>GCGCCCAGAAATTCAACGGCCTGACAGTTCTCCCACCTCTGCTGACCGACGAGA<br>TGATCGCTCAGTACACCTCTGCCCTGCTGGCTGGCACCATCACATCTGGGTGGA<br>CATTTGGCGCCGGCGCCGCCCTGCAGATCCCCTTTGCCATGCAGATGGCCTATA<br>GATTCAACGGAATCGGCGTGACCCAGAACGTGCTGTATGAAAACCAGAAGCTGA<br>TCGCTAACCAGTTCAATTCTGCCATCGGCAAGATCCAGGACTCCCTCTCCTCTAC

	CGCCAGCGCCCTGGGCAAACTGCAGGACGTGGTGAATCAGAACGCCCAAGCCC TGAACACCCTGGTGAAGCAGCTCAGCAGCAATTTTGGCGCCATCAGCTCTGTGC TGAACGATATCCTGTCTAGACTGGACCCTCCAGAAGCCGAAGTCCAGATCGATA GACTGATCACAGGCAGACTGCAGTCCCTGCAAACCTACGTGACCCAACAGCTGA TCAGGGCCGCTGAAATAAGAGCCAGCGCCAATCTCGCCGCTACCAAGATGTCCG AGTGTGTGCTGGGACAGTCTAAACGCGTTGACTTCTGCGGCAAAGGCTATCACC TGATGAGCTTCCCCCAGAGCGCGCCGCACGGCGTGGTGTTCCTGCATGTGACAT ACGTGCCTGCCCAAGAGAAGAATTTCACAACCGCCCCTGCCATCTGCCACGACG GCAAGGCCCACTTCCCAAGAGAGGGCGTTTTCGTTTCCAATGGCACACACTGGT TCGTGACACAAAGAAACTTCTACGAACCCCAGATTATCACCACCGACAACACCTT CGTGAGTGGCAATTGTGACGTGGTCATCGGAATCGTGAACAACACAGTGTACGA CCCTCTGCAACCTGAGCTGGACTCTTTTAAGGAAGAGCTGGACAAGTACTTTAAA AACCACACCAGCCCCGATGTGGACCTGGGCGACATCAGTGGCATTAACGCCAGC GTGGTGAACATCCAAAAGGAAATCGACAGACTGAACGAGGTGGCCAAGAACCTG AACGAGTCCCTGATCGACCTGCAGGAGCTCGGCAAATACGAGCAGGGATCCGG ATACATCCCCGAGGCCCCCAGAGATGGCCAGGCCTACGTGCGGAAGGACGGCG AGTGGGTACTGCTGAGCACATTCCTGGGCAGATCCCTGGAGGTGCTGTTCCAGG GCCCAGGCCATCACCACCATCACCACCATCATAGCGCCTGGTCCCACCCCCAGT TCGAGAAGGGCGGCGGTAGTGGAGGGGGCGGATCTGGCGGCTCAGCTTGGAG CCACCCCCAGTTCGAAAAGTGA
SARS-CoV-2 RBD-Fc	ATGCGGCCTACTTGGGCCTGGTGGCTGTTCCTGGTGCTGCTGCTAGCTCTGTGG GCACCCGCTAGAGGGCGGGTGCAGCCTACAGAGAGCATCGTGCGATTTCCAAA CATTACCAACCTCTGCCCCTTCGGCGAGGTGTTTAACGCCACAAGATTTGCCTCC GTTTACGCCTGGAATAGAAAGAGAATCAGCAATTGTGTGGCCGACTACTCCGTG CTGTATAACAGCGCCTCTTTCAGCACCTTCAAGTGCTACGGCGTTTCCCCAACAA AGCTGAATGACCTGTGCTTCACCAACGTGTACGCCGACTCCTTCGTAATTAGAGG CGATGAGGTGCGGCAGATCGCACCAGGCCAGACCGGTAAGATCGCTGACTACA ACTATAAGCTGCCTGATGATTTTACAGGCTGCGTGATCGCCTGGAACTCTAACAA CCTGGATAGCAAGGTGGGCGGCAACTACAACTACCTGTACCGGCTGTTTCGCAA GTCTAACCTGAAACCTTTCGAGAGAGACATCTCCACAGAGATCTACCAGGCCGG TTCTACACCTTGTAACGGGGTGGAAGGCTTCAACTGTTACTTCCCTCTGCAAAGC TACGGCTTCCAGCCTACCAATGGAGTCGGCTACCAGCCATACCGGGTGGTCGTG CTGTCCTTCGAGTTACTCCACGCCCCCGCCACCGTCTGCGGTCCTAAGAAGTCC ACCAATCTGGTTAAGAACAAATGCGTGAACTTCAACTTCAACGGCCTGACCGGGA CCGGCGTGCTGACCGAAAGCAACAAAAAGTTCCTCCCCTTCCAGCAGTTCGGCC GTGATATCGCTGACACCACAGATGCCGTCAGAGATCCACAGACCCTGGAAATCC TGGATATTACACCCTGCTCCGGATCCCTGGAGGTGCTGTTCCAGGGCCCAACTC ACACATCCCCACCGAGCCCAGCACCTGAACTCCTGGGGGGACCGTCAGTCTTCC TCTTCCCCCCAAAACCCAAGGACACCCTCATGATCTCCCGGACCCCTGAGGTCA CATGCGTGGTGGTGGACGTGAGCCACGAAGACCCTGAGGTCAAGTTCAACTGGT ACGTGGACGGCGTGGAGGTGCATAATGCCAAGACAAAGCCGCGGGAGGAGCAG TACAACAGCACGTACCGTGTGGTCAGCGTCCTCACCGTCCTGCACCAGGACTGG CTGAATGGCAAGGAGTACAAGTGCAAGGTCTCCAACAAAGCCCTCCCAGCCCCC ATCGAGAAAACCATCTCCAAAGCCAAAGGGCAGCCCCGAGAACCACAGGTGTAC ACCAAACCCCCATCCCGGGATGAGCTGACCAAGAACCAGGTCAGCCTGTCCTGC CTGGTCAAAGGCTTCTATCCCAGCGACATCGCCGTGGAGTGGGAGAGCAATGG GCAGCCGGAGAACAACTACAAGACCACGGTCCCCGTGCTGGACTCCGACGGCT CCTTCAGGCTCGCCAGCTATCTCACCGTGGACAAGAGCAGGTGGCAGCAGGGG AACGTCTTCTCATGCTCCGTGATGCATGAGGCTCTGCACAACCACTACACGCAGA AGAGCCTCTCCCTGTCTCCGGGTAAACATCACCATCATCACCACCATCACTAG
SARS-CoV (S-2P)	ATGTTCATCTTCCTGCTGTTCCTGACCCTGACCAGCGGCAGCGACCTGGACCGG TGCACCACCTTCGACGACGTGCAGGCCCCCAACTACACCCAGCACACCAGCAGC ATGCGGGGCGTGTACTACCCCGACGAGATCTTCCGGAGCGACACCCTGTACCTG ACCCAGGACCTGTTCCTGCCCTTCTACAGCAACGTGACCGGCTTCCACACCATC AACCACACCTTCGGCAACCCCGTGATCCCCTTCAAGGACGGCATCTACTTCGCC GCCACCGAGAAGAGCAACGTGGTGCGGGGCTGGGTGTTCGGCAGCACCATGAA

	CAACAAGAGCCAGAGCGTGATCATCATCAACAACAGCACCAACGTGGTGATCCG<br>GGCCTGCAACTTCGAGCTGTGCGACAACCCCTTCTTCGCCGTGAGCAAGCCCAT<br>GGGCACCCAGACCCACACCATGATCTTCGACAACGCCTTCAACTGCACCTTCGA<br>GTACATCAGCGACGCCTTCAGCCTGGACGTGAGCGAGAAGAGCGGCAACTTCAA<br>GCACCTGCGGGAGTTCGTGTTCAAGAACAAGGACGGCTTCCTGTACGTGTACAA<br>GGGCTACCAGCCCATCGACGTGGTGCGGGACCTGCCCAGCGGCTTCAACACCC<br>TGAAGCCCATCTTCAAGCTGCCCCTGGGCATCAACATCACCAACTTCCGGGCCA<br>TCCTGACCGCCTTCAGCCCCGCCCAGGACATCTGGGGCACCAGCGCCGCCGCC<br>TACTTCGTGGGCTACCTGAAGCCCACCACCTTCATGCTGAAGTACGACGAGAAC<br>GGCACCATCACCGACGCCGTGGACTGCAGCCAGAACCCCCTGGCCGAGCTGAA<br>GTGCAGTGTGAAGAGCTTCGAGATCGACAAGGGCATCTACCAGACCAGCAACTT<br>CCGGGTGGTGCCCAGCGGCGACGTGGTGCGGTTCCCCAACATCACCAACCTGT<br>GCCCCTTCGGCGAGGTGTTCAACGCCACCAAGTTCCCCAGCGTGTACGCCTGG<br>GAGCGGAAGAAGATCAGCAACTGCGTGGCCGACTACAGCGTGCTGTACAACAG<br>CACCTTCTTCAGCACCTTCAAGTGCTACGGCGTGAGCGCCACCAAGCTGAACGA<br>CCTGTGCTTCAGCAACGTGTACGCCGACAGCTTCGTGGTGAAGGGCGACGACGT<br>GCGGCAGATCGCCCCCGGCCAGACCGGCGTGATCGCCGACTACAACTACAAGC<br>TGCCCGACGACTTCATGGGCTGCGTGCTGGCCTGGAACACCCGGAACATCGAC<br>GCCACCAGCACCGGCAACTACAACTACAAGTACCGGTACCTGCGGCACGGCAA<br>GCTGCGGCCCTTCGAGCGGGACATCAGCAACGTGCCCTTCAGCCCCGACGGCA<br>AGCCCTGCACCCCCCCCGCCCTGAACTGCTACTGGCCCCTGAACGACTACGGCT<br>TCTACACCACTACCGGCATCGGCTACCAGCCCTACCGGGTGGTGGTGCTGAGCT<br>TCGAGCTGCTGAACGCCCCCGCCACCGTGTGCGGCCCCAAGCTGAGCACCGAC<br>CTGATCAAGAACCAGTGCGTGAACTTCAACTTCAACGGCCTGACCGGCACCGGC<br>GTGCTGACCCCCAGCAGCAAGCGGTTCCAGCCCTTCCAGCAGTTCGGCCGGGA<br>CGTGAGCGACTTCACCGACAGCGTGCGGGACCCCAAGACCAGCGAGATCCTGG<br>ACATCAGCCCCTGCGCCTTCGGCGGCGTGAGCGTGATCACCCCCGGTACCAAC<br>GCCAGCAGCGAGGTGGCCGTGCTGTACCAGGACGTGAACTGCACCGACGTGAG<br>CACCGCCATCCACGCCGACCAGCTGACCCCCGCCTGGCGGATCTACAGCACCG<br>GCAACAACGTGTTCCAGACCCAGGCCGGCTGCCTGATCGGCGCCGAGCACGTG<br>GACACCAGCTACGAGTGCGACATCCCCATCGGCGCCGGCATCTGCGCCAGCTA<br>CCACACCGTGAGCCTGCTGCGGAGCACCAGCCAGAAGAGCATCGTGGCCTACA<br>CCATGAGCCTGGGCGCCGACAGCAGCATCGCCTACAGCAACAACACCATCGCC<br>ATCCCCACCAACTTCAGCATCAGCATCACCACCGAGGTGATGCCCGTGAGCATG<br>GCCAAGACCAGCGTGGACTGCAACATGTACATCTGCGGCGACAGCACCGAGTG<br>CGCCAACCTGCTGCTGCAGTACGGCAGCTTCTGCACCCAGCTGAACCGGGCCC<br>TGAGCGGCATCGCCGCCGAGCAGGACCGGAACACCCGGGAGGTGTTCGCCCA<br>GGTGAAGCAGATGTACAAGACCCCCACCCTGAAGTACTTCGGCGGCTTCAACTT<br>CAGCCAGATCCTGCCCGACCCCCTGAAGCCCACCAAGCGGAGCTTCATCGAGG<br>ACCTGCTGTTCAACAAGGTGACCCTGGCCGACGCCGGCTTCATGAAGCAGTACG<br>GCGAGTGCCTGGGCGACATCAACGCCCGGGACCTGATCTGCGCCCAGAAGTTC<br>AACGGCCTGACCGTGCTGCCCCCCCTGCTGACCGACGACATGATCGCCGCCTA<br>CACCGCCGCCCTGGTGAGCGGCACCGCCACCGCCGGCTGGACCTTCGGCGCC<br>GGCGCCGCCCTGCAGATCCCCTTCGCCATGCAGATGGCCTACCGGTTCAACGG<br>CATCGGCGTGACCCAGAACGTGCTGTACGAGAACCAGAAGCAGATCGCCAACCA<br>GTTCAACAAGGCCATCAGCCAGATCCAGGAGAGCCTGACCACCACCAGCACCGC<br>CCTGGGCAAGCTGCAGGACGTGGTGAACCAGAACGCCCAGGCCCTGAACACCC<br>TGGTGAAGCAGCTGAGCTCCAACTTCGGCGCCATCAGCAGCGTGCTGAACGACA<br>TCCTGAGCCGGCTGGACCCCCCCGAGGCCGAGGTGCAGATCGACCGGCTGATC<br>ACCGGCCGGCTGCAGAGCCTGCAGACCTACGTGACCCAGCAGCTGATCCGGGC<br>CGCCGAGATCCGGGCCAGCGCCAACCTGGCCGCCACCAAGATGAGCGAGTGCG<br>TGCTGGGCCAGAGCAAGCGGGTGGACTTCTGCGGCAAGGGCTACCACCTGATG<br>AGCTTCCCCCAGGCCGCCCCCCACGGCGTGGTGTTCCTGCACGTGACCTACGT<br>GCCCAGCCAGGAGCGGAACTTCACCACCGCCCCCGCCATCTGCCACGAGGGCA<br>AGGCCTACTTCCCCCGGGAGGGCGTGTTCGTGTTCAACGGCACCAGCTGGTTCA<br>TCACCCAGCGGAACTTCTTCAGCCCCCAGATCATCACCACCGACAACACCTTCGT

	GAGCGGCAACTGCGACGTGGTGATCGGCATCATCAACAACACCGTGTACGACCC<br>CCTGCAGCCCGAGCTGGACAGCTTCAAGGAGGAGCTGGACAAGTACTTCAAGAA<br>CCACACCAGCCCCGACGTGGACCTGGGCGACATCAGCGGCATCAACGCCAGCG<br>TGGTGAACATCCAGAAGGAGATCGACCGGCTGAACGAGGTGGCCAAGAACCTG<br>AACGAGAGCCTGATCGACCTGCAGGAGCTGGGCAAGTACGAGCAGGGATCCGG<br>ATACATCCCCGAGGCCCCCAGAGATGGCCAGGCCTACGTGCGGAAGGACGGCG<br>AGTGGGTACTGCTGAGCACATTCCTGGGCAGATCCCTGGAGGTGCTGTTCCAGG<br>GCCCAGGCCACCACCACCACCACCACCATCATAGCGCCTGGTCCCACCCCCAGT<br>TCGAGAAGTGA
MERS (S-2P)	ATGATTCACTCCGTGTTCCTGCTGATGTTCCTGCTGACTCCTACAGAGAGCTATG<br>TGGATGTGGGACCTGATTCCGTCAAGAGCGCCTGCATCGAAGTGGACATTCAGC<br>AGACCTTCTTTGATAAGACATGGCCAAGACCCATCGACGTGAGCAAAGCCGATG<br>GCATCATCTACCCTCAGGGGAGGACCTATTCCAATATCACAATTACTTACCAGGG<br>CCTGTTCCCATATCAGGGAGACCACGGCGATATGTACGTGTATTCTGCTGGCCAT<br>GCAACAGGGACCACACCTCAGAAGCTGTTTGTGGCTAACTACAGCCAGGACGTC<br>AAACAGTTCGCAAATGGATTTGTGGTCCGCATCGGCGCCGCTGCAAACTCTACC<br>GGCACAGTGATCATTTCACCTAGCACTTCCGCAACCATCCGAAAAATCTACCCAG<br>CCTTCATGCTGGGAAGCTCCGTGGGCAATTTTAGCGACGGAAAATGGGACGGT<br>TCTTTAACCACACCCTGGTGCTGCTGCCTGATGGATGCGGCACACTGCTGAGGG<br>CTTTCTACTGTATCCTGGAGCCACGCAGCGGAAACCACTGCCCCGCAGGAAATA<br>GCTACACCTCCTTTGCCACATATCATACTCCAGCTACCGACTGTTCCGATGGCAA<br>CTACAATCGAAACGCCTCTCTGAATAGTTTCAAGGAATACTTCAACCTGCGGAAT<br>TGCACATTCATGTACACTTATAACATCACCGAGGACGAAATTCTGGAGTGGTTCG<br>GAATCACTCAGACCGCACAGGGCGTGCACCTGTTTTCTAGTCGCTACGTCGACC<br>TGTATGGCGGGAACATGTTCCAGTTTGCCACTCTGCCCGTGTACGATACCATCAA<br>GTACTATTCCATCATTCCTCATTCAATCCGCAGCATTCAGTCCGATCGAAAGGCTT<br>GGGCCGCTTTCTACGTGTATAAACTGCAGCCACTGACCTTCCTGCTGGACTTTAG<br>CGTCGATGGCTACATCCGGAGAGCCATTGACTGCGGGTTTAATGATCTGTCCCA<br>GCTGCACTGTTCTTACGAAAGTTTCGACGTGGAGTCCGGCGTGTATTCTGTCTCA<br>AGCTTTGAGGCCAAGCCCTCTGGGAGTGTGGTCGAGCAGGCTGAAGGAGTGGA<br>GTGCGATTTCAGTCCTCTGCTGTCAGGGACCCCCCCTCAGGTGTACAACTTCAA<br>GCGGCTGGTCTTTACTAACTGTAACTACAATCTGACCAAGCTGCTGTCACTGTTC<br>AGCGTGAATGACTTTACATGCTCCCAGATCAGCCCCGCAGCCATTGCTAGTAACT<br>GTTACTCCTCTCTGATCCTGGACTACTTCTCATATCCACTGAGTATGAAGAGCGA<br>CCTGAGCGTGAGTTCAGCCGGCCCCATCAGCCAGTTCAACTATAAACAGAGCTT<br>CAGCAATCCTACATGCCTGATTCTGGCTACTGTGCCACATAATCTGACTACCATC<br>ACTAAGCCCCTGAAATACTCCTATATTAACAAGTGCAGCCGGTTCCTGTCCGACG<br>ATAGAACCGAAGTGCCACAGCTGGTCAACGCCAATCAGTACTCTCCCTGTGTGA<br>GTATCGTCCCTTCAACCGTGTGGGAAGACGGGGATTACTATAGAAAACAGCTGA<br>GCCCCCTGGAGGGAGGAGGATGGCTGGTGGCATCCGGATCTACAGTCGCCATG<br>ACTGAGCAGCTGCAGATGGGGTTCGGAATCACAGTGCAGTACGGCACAGACACT<br>AACTCTGTCTGTCCCAAGCTGGAATTCGCTAACGATACTAAGATCGCAAGTCAGC<br>TGGGAAACTGCGTGGAGTACTCTCTGTATGGCGTGAGTGGCAGAGGGGTCTTCC<br>AGAATTGTACCGCAGTGGGCGTCCGACAGCAGCGGTTTGTGTACGACGCCTATC<br>AGAATCTGGTCGGCTACTATAGCGACGATGGGAACTACTATTGCCTGAGGGCCT<br>GTGTGAGCGTCCCTGTGTCCGTCATCTACGATAAGGAAACCAAAACACACGCCA<br>CACTGTTCGGGTCCGTGGCTTGCGAGCATATTAGCTCCACAATGTCTCAGTACAG<br>TAGATCAACTAGGTCAATGCTGAAGAGGCGCGATAGCACCTATGGACCTCTGCA<br>GACACCAGTGGGGTGTGTCCTGGGACTGGTGAACTCTAGTCTGTTTGTCGAGGA<br>CTGCAAGCTGCCCCTGGGCCAGAGCCTGTGCGCCCTGCCCGACACCCCCAGCA<br>CCCTGACCCCCGCCAGCGTGGGCAGCGTGCCCGGCGAGATGCGGCTGGCCAG<br>CATCGCCTTCAACCACCCCATCCAGGTGGACCAGCTGAACAGCAGCTACTTCAA<br>GCTGAGCATCCCCACCAACTTCAGCTTCGGCGTGACCCAGGAGTACATCCAGAC<br>CACCATCCAGAAGGTGACCGTGGACTGCAAGCAGTACGTGTGCAACGGCTTCCA<br>GAAGTGCGAGCAGCTGCTGCGGGAGTACGGCCAGTTCTGCAGCAAGATCAACC<br>AGGCCCTGCACGGCGCCAACCTGCGGCAGGACGACAGCGTGCGGAACCTGTTC

	GCTAGCGTGAAGAGCAGCCAGAGCAGCCCCATCATCCCCGGCTTCGGCGGCGA CTTCAACCTGACCCTGCTGGAGCCCGTGAGCATCAGCACCGGCAGCCGGAGCG CCCGGAGCGCCATCGAGGACCTGCTGTTCGACAAGGTGACCATCGCCGACCCC GGCTACATGCAGGGCTACGACGACTGCATGCAGCAGGGCCCCGCCAGCGCCCG GGACCTGATCTGCGCCCAGTACGTGGCCGGCTACAAGGTGCTGCCCCCCCTGA TGGACGTGAACATGGAGGCCGCCTACACCAGCAGCCTGCTGGGCAGCATCGCC GGCGTGGGCTGGACCGCCGGCCTGAGCAGCTTCGCCGCCATCCCCTTCGCCCA GAGCATCTTCTACCGGCTGAACGGCGTGGGCATCACCCAGCAGGTGCTGAGCG AGAACCAGAAGCTGATCGCCAACAAGTTCAACCAGGCCCTGGGCGCCATGCAGA CCGGCTTCACCACCACCAACGAGGCCTTCCACAAGGTGCAGGACGCCGTGAAC AACAACGCCCAGGCCCTGAGCAAGCTGGCCAGCGAGCTGAGCAACACCTTCGG CGCCATCAGCGCCAGCATCGGCGACATCATCCAGCGGCTGGACCCCCCCGAGC AGGACGCCCAGATCGACCGGCTGATCAACGGCCGGCTGACCACCCTGAACGCC TTCGTGGCCCAGCAGCTGGTGCGGAGCGAGAGCGCCGCCCTGAGCGCCCAGCT GGCCAAGGACAAGGTGAACGAGTGCGTGAAGGCCCAGAGCAAGCGGAGCGGCT TCTGCGGCCAGGGCACCCACATCGTGAGCTTCGTGGTGAACGCCCCCAACGGC CTGTACTTCATGCACGTGGGCTACTACCCCAGCAACCACATCGAGGTGGTGAGC GCCTACGGCCTGTGCGACGCCGCCAACCCCACCAACTGCATCGCCCCCGTGAA CGGCTACTTCATCAAGACCAACAACACCCGGATCGTGGACGAGTGGAGCTACAC CGGCAGCAGCTTCTACGCCCCCGAGCCCATCACCAGCCTGAACACCAAGTACGT GGCCCCCCAGGTGACCTACCAGAACATCAGCACCAACCTGCCCCCCCCCCTGCT GGGCAACAGCACCGGCATCGACTTCCAGGACGAGCTGGACGAGTTCTTCAAGAA CGTGAGCACCAGCATCCCCAACTTCGGCAGCCTGACCCAGATCAACACCACCCT GCTGGACCTGACCTACGAGATGCTGAGCCTGCAGCAGGTGGTGAAGGCCCTGA ACGAGAGCTACATCGACCTGAAGGAGCTGGGCAACTACACCTACGGATCCGGAT ACATCCCCGAGGCCCCCAGAGATGGCCAGGCCTACGTGCGGAAGGACGGCGAG TGGGTACTGCTGAGCACATTCCTGGGCAGATCCCTGGAGGTGCTGTTCCAGGGC CCAGGCCATCACCACCATCACCACCATCATAGCGCCTGGTCCCACCCCCAGTTC GAGAAGGGCGGCGGTAGTGGAGGGGGCGGATCTGGCGGCTCAGCTTGGAGCC ACCCCCAGTTCGAAAAGTGA
229E (S-2P)	ATGTTTGTGCTGCTGGTGGCCTATGCACTGCTGCACATCGCAGGATGCCAGACC ACAAACGGCACCAATACAAGCCACTCCGTGTGCAACGGATGCGTGGGACACAGC GAGAACGTGTTCGCAGTGGAGAGCGGAGGATACATCCCATCCAATTTCGCCTTT AACAATTGGTTCCTGCTGACCAACACAAGCTCCGTGGTGGACGGCGTGGTGAGA TCCTTTCAGCCTCTGCTGCTGAACTGCCTGTGGTCTGTGAGCGGCTCCCAGTTCA CCACAGGCTTCGTGTACTTTAATGGCACCGGCAGAGGCGCCTGTAAGGGCTTCT ACTCTAACGCCTCTAGCGACGTGATCAGGTATAACATCAATTTTGAGGAGAATCT GCGGAGAGGCACAATCCTGTTTAAGACCTCCTACGGCGCCGTGGTGTTCTATTG CACAAACAATACCCTGGTGTCCGGCGATGCCCACATCCCATCTGGCACCGTGCT GGGCAACTTCTATTGTTTTGTGAACACCACAATCGGCAATGAGACCACAAGCGCC TTTGTGGGCGCCCTGCCAAAGACCGTGCGCGAGTTCGTGATCAGCCGGACAGG CCACTTTTACATCAATGGCTACAGGTATTTCTCCCTGGGCGACGTGGAGGCCGT GAACTTCAATGTGACCAACGCCGCCACCACAGTGTGCACAGTGGCCCTGGCCAG CTATGCCGATGTGCTGGTGAACGTGTCCCAGACCGCCATCGCCAATATCATCTA CTGCAACTCCGTGATCAATCGGCTGAGATGTGACCAGCTGTCTTTCGACGTGCC TGATGGCTTTTATTCTACAAGCCCCATCCAGCCTGTGGAGCTGCCCGTGAGCATC GTGAGCCTGCCAGTGTACCACAAGCACACCTTCATCGTGCTGTATGTGAATTTTG AGCACAGGAGGGGACCTGGCAAGTGCTACAACTGTCGCCCTGCCGTGATCAATA TCACCCTGGCCAACTTCAATGAGACAAAGGGCCCTCTGTGCGTGGACACAAGCC ACTTCACCACACAGTTTGTGGATAACGTGAAGCTGGCCCGGTGGTCCGCCTCTA TCAACACCGGCAATTGTCCATTCTCCTTTGGCAAGGTGAACAATTTCGTGAAGTT TGGCAGCGTGTGCTTTAGCCTGAAGGATATCCCAGGCGGCTGTGCCATGCCCAT CATGGCCAACCTGGTGAATAGCAAGTCCCACAATATCGGCAGCCTGTACGTGTC TTGGAGCGACGGCGATGTGATCACAGGCGTGCCTAAGCCAGTGGAGGGCGTGT CCTCTTTCATGAACGTGACACTGAATAAGTGCACCAAGTACAACATCTATGACGT GAGCGGCGTGGGCGTGATCCGCATCTCCAACGATACCTTTCTGAATGGCATCAC

	CTATACATCCACCTCTGGCAATCTGCTGGGCTTCAAGGACGTGACAAACGGCAC<br>CATCTACAGCATCACACCATGTAATCCCCCTGATCAGCTGGTGGTGTATCAGCAG<br>GCAGTGGTGGGAGCAATGCTGTCCGAGAACTTCACCTCCTACGGCTTTTCTAAT<br>GTGGTGGAGATGCCCAAGTTCTTTTACGCCTCTAACGGCACATATAATTGCACCG<br>ACGCCGTGCTGACATATAGCAGCTTCGGCGTGTGCGCCGATGGCTCTATCATCG<br>CCGTGCAGCCTCGGAACGTGAGCTACGACAGCGTGTCCGCCATCGTGACCGCC<br>AACCTGTCTATCCCTTTCAATTGGACCACAAGCGTGCAGGTGGAGTACCTGCAGA<br>TCACATCCACCCCAATCGTGGTGGACTGCTCTACCTACGTGTGCAACGGCAATG<br>TGCGCTGCGTGGAGCTGCTGAAGCAGTACACAAGCGCCTGTAAGACCATCGAG<br>GATGCCCTGCGGAACTCCGCCATGCTGGAGAGCGCCGACGTGTCCGAGATGCT<br>GACCTTCGATAAGAAGGCCTTTACACTGGCCAACGTGTCTAGCTTTGGCGACTAT<br>AATCTGTCCTCTGTGATCCCATCTCTGCCACGGTCTGGCAGCAGAGTGGCAGGC<br>AGGAGCGCCATCGAGGATATCCTGTTCTCTAAGCTGGTGACAAGCGGACTGGGC<br>ACCGTGGACGCCGATTACAAGAAGTGCACCAAGGGACTGAGCATCGCAGACCTG<br>GCATGTGCCCAGTACTATAACGGAATCATGGTGCTGCCAGGAGTGGCAGATGCA<br>GAGCGCATGGCCATGTATACAGGCTCCCTGATCGGCGGCATCGCCCTGGGAGG<br>ACTGACCTCCGCCGCATCTATCCCTTTCTCCCTGGCCATCCAGTCTCGCCTGAAT<br>TACGTGGCCCTGCAGACCGACGTGCTGCAGGAGAACCAGAGAATCCTGGCCGC<br>CAGCTTCAACAAGGCCATGACCAATATCGTGGACGCCTTTACAGGCGTGAATGAT<br>GCCATCACACAGACCTCCCAGGCCCTGCAGACAGTGGCAACCGCCCTGAACAA<br>GATCCAGGATGTGGTGAATCAGCAGGGCAACTCTCTGAATCACCTGACCAGCCA<br>GCTGAGACAGAACTTTCAGGCCATCAGCTCCTCTATCCAGGCCATCTACGACAG<br>GCTGGACCCCCCACAGGCAGACCAGCAGGTGGATAGACTGATCACAGGCAGGC<br>TGGCCGCCCTGAACGTGTTCGTGAGCCACACACTGACCAAGTATACCGAGGTGA<br>GGGCATCTAGGCAGCTGGCACAGCAGAAGGTGAACGAGTGCGTGAAGTCCCAG<br>TCTAAGAGATACGGCTTTTGTGGCAATGGCACCCACATCTTCAGCCTGGTGAACG<br>CAGCACCAGAGGGACTGGTGTTCCTGCACACAGTGCTGCTGCCCACCCAGTACA<br>AGGACGTGGAGGCATGGTCCGGACTGTGCGTGGATGGCATCAACGGCTACGTG<br>CTGAGGCAGCCAAATCTGGCCCTGTATAAGGAGGGCAACTACTATCGCATCACC<br>AGCCGGATCATGTTTGAGCCTAGAATCCCAACAATCGCCGACTTCGTGCAGATC<br>GAGAACTGTAATGTGACCTTTGTGAACATCAGCAGGTCCGAGCTGCAGACAATC<br>GTGCCCGAGTACATCGACGTGAATAAGACCCTGCAGGAGCTGTCCTACAAGCTG<br>CCCAACTATACCGTGCCTGATCTGGTGGTGGAGCAGTATAACCAGACAATCCTG<br>AATCTGACCAGCGAGATCTCCACACTGGAGAACAAGTCTGCCGAGCTGAATTAC<br>ACCGTGCAGAAGCTGCAGACACTGATCGACAACATCAATAGCACACTGGTGGAT<br>CTGAAGTGGCTGAACCGGGTGGAGACCGGATCCGGATACATCCCCGAGGCCCC<br>CAGAGATGGCCAGGCCTACGTGCGGAAGGACGGCGAGTGGGTACTGCTGAGCA<br>CATTCCTGGGCAGATCCCTGGAGGTGCTGTTCCAGGGCCCAGGCCATCACCACC<br>ATCACCACCATCATAGCGCCTGGTCCCACCCCCAGTTCGAGAAGGGCGGCGGTA<br>GTGGAGGGGGCGGATCTGGCGGCTCAGCTTGGAGCCACCCCCAGTTCGAAAAG<br>TGA
HKU1 (S-2P)	ATGTTTCTGATTATCTTTATTCTGCCCACTACCCTGGCCGTCATTGGAGACTTCAA<br>CTGCACTAACTCATTCATTAACGACTACAACAAGACAATCCCCCGCATTTCCGAG<br>GATGTGGTCGACGTGTCTCTGGGGCTGGGAACATACTATGTGCTGAATCGAGTC<br>TATCTGAACACCACACTGCTGTTTACTGGATACTTCCCTAAATCCGGCGCCAACT<br>TCCGCGACCTGGCTCTGAAGGGCTCAATCTACCTGAGCACCCTGTGGTACAAAC<br>CCCCTTTTCTGTCAGATTTCAACAATGGGATCTTTAGCAAGGTGAAGAACACTAA<br>GCTGTACGTCAACAACACCCTGTACTCTGAATTCAGTACAATCGTGATTGGATCT<br>GTCTTTGTGAACACTAGTTATACCATCGTGGTCCAGCCTCACAATGGCATCCTGG<br>AGATTACCGCCTGTCAGTATACAATGTGCGAATACCCACACACCGTGTGCAAGTC<br>AAAAGGCAGCATTAGAAACGAGTCCTGGCATATCGATAGCTCCGAACCCCTGTG<br>CCTGTTTAAGAAAAACTTCACATACAACGTGAGCGCCGACTGGCTGTATTTCCAC<br>TTTTACCAGGAGAGGGGAGTGTTCTATGCCTACTATGCTGATGTCGGCATGCCAA<br>CTACCTTCCTGTTTTCCCTGTACCTGGGCACAATTCTGTCTCATTACTATGTGATG<br>CCCCTGACTTGCAACGCCATCTCTAGTAACACCGACAATGAGACACTGGAATATT<br>GGGTGACCCCTCTGTCCCGGAGACAGTACCTGCTGAACTTCGATGAGCACGGC

	GTGATTACAAATGCTGTCGACTGTTCAAGCTCCTTCCTGAGTGAAATCCAGTGCA<br>AGACTCAGTCATTTGCACCTAACACCGGGGTGTATGATCTGTCTGGCTTCACCGT<br>CAAACCAGTGGCCACTGTCTACAGGCGCATTCCAAACCTGCCCGACTGTGATAT<br>CGACAATTGGCTGAACAATGTCTCCGTGCCATCTCCCCTGAATTGGGAGCGACG<br>GATCTTCAGCAACTGCAATTTCAACCTGTCAACTCTGCTGAGACTGGTCCATGTG<br>GATTCATTCAGCTGTAACAATCTGGACAAGAGCAAAATCTTTGGCTCCTGCTTCA<br>ACTCTATTACCGTGGACAAGTTCGCCATCCCAAATAGAAGGCGCGACGATCTGC<br>AGCTGGGGTCTAGTGGATTTCTGCAGTCAAGCAACTACAAGATCGATATTTCCTC<br>TAGTTCATGCCAGCTGTACTATAGCCTGCCACTGGTCAATGTGACCATCAACAAT<br>TTCAATCCCAGCTCCTGGAACCGACGGTACGGCTTCGGGTCCTTTAACCTGTCTA<br>GTTATGATGTGGTCTACTCTGACCACTGTTTTAGTGTGAACTCAGATTTCTGTCCT<br>TGCGCAGACCCAAGTGTGGTCAATTCATGCGCCAAGAGCAAACCACCCTCCGCT<br>ATTTGTCCTGCAGGCACAAAGTATAGGCATTGCGATCTGGACACAACTCTGTACG<br>TGAAAAACTGGTGTCGCTGCAGCTGTCTGCCTGACCCAATCAGCACTTACTCCCC<br>CAATACCTGCCCTCAGAAGAAAGTGGTCGTGGGAATTGGCGAGCACTGTCCTGG<br>GCTGGGAATCAACGAGGAAAAGTGCGGGACCCAGCTGAATCATTCAAGCTGCTT<br>CTGTTCCCCAGATGCCTTTCTGGGATGGTCTTTCGACAGTTGTATTTCAAACAAC<br>AGGTGCAACATCTTTTCTAATTTCATCTTTAACGGGATTAATAGTGGAACCACATG<br>TTCAAACGATCTGCTGTACAGCAATACTGAGATCAGCACCGGCGTCTGCGTGAA<br>CTATGACCTGTACGGGATCACAGGCCAGGGGATTTTCAAGGAAGTGAGCGCCGC<br>TTACTATAACAATTGGCAGAACCTGCTGTATGATAGCAATGGCAACATCATTGGG<br>TTCAAGGACTTTCTGACCAACAAGACATATACTATCCTGCCCTGCTACTCTGGCA<br>GAGTGAGTGCAGCCTTCTACCAGAACTCCTCTAGTCCTGCCCTGCTGTATAGGAA<br>TCTGAAGTGTAGCTACGTGCTGAACAATATCAGCTTTATTTCCCAGCCATTCTATT<br>TTGACAGCTACCTGGGCTGCGTGCTGAACGCTGTCAATCTGACCTCCTACAGCG<br>TGAGCAGCTGTGATCTGAGAATGGGATCCGGCTTCTGCATTGACTATGCACTGC<br>CATCCAGCGGGGGGAGCGGAAGCGGAATCAGTTCACCATACAGGTTCGTGACTT<br>TTGAGCCCTTCAACGTGAGCTTCGTGAACGACAGTGTGGAGACCGTCGGCGGG<br>CTGTTTGAAATCCAGATTCCAACAAACTTCACTATTGCAGGCCACGAGGAGTTCA<br>TCCAGACCAGCTCCCCCAAAGTGACAATCGATTGTTCAGCCTTCGTCTGCAGCAA<br>TTATGCTGCATGTCATGATCTGCTGAGCGAGTACGGAACTTTTTGCGACAATATC<br>AACAGCATTCTGAACGAAGTGAATGATCTGCTGGACATTACCCAGCTGCAGGTG<br>GCTAACGCACTGATGCAGGGCGTCACACTGAGCTCTAATCTGAACACTAATCTGC<br>ACAGCGATGTGGACAATATCGACTTCAAGAGCCTGCTGGGATGTCTGGGGTCCC<br>AGTGCGGATCAAGCTCCCGCTCCCTGCTGGAGGATCTGCTGTTCAACAAGGTGA<br>AACTGTCTGACGTGGGCTTTGTCGAGGCCTACAACAATTGTACCGGAGGCAGCG<br>AAATCCGGGATCTGCTGTGCGTGCAGAGCTTCAATGGCATCAAAGTCCTGCCTC<br>CAATTCTGTCCGAAACTCAGATCAGCGGGTATACTACCGCAGCTACCGTGGCAG<br>CAATGTTCCCACCTTGGAGCGCCGCAGCAGGAGTGCCCTTTTCCCTGAACGTCC<br>AGTACCGCATCAATGGGCTGGGAGTGACCATGGACGTGCTGAACAAGAATCAGA<br>AACTGATTGCCAACGCTTTCAATAAGGCTCTGCTGAGCATCCAGAACGGGTTTAC<br>CGCAACAAATTCTGCACTGGCCAAAATCCAGAGTGTCGTGAACGCTAATGCACA<br>GGCCCTGAACAGCCTGCTGCAGCAGCTGTTTAATAAGTTCGGAGCTATTTCTAGT<br>TCACTGCAGGAGATCCTGTCTAGACTGGATCCCCCCGAAGCACAGGTGCAGATC<br>GACCGGCTGATTAACGGCAGACTGACCGCACTGAATGCCTACGTGTCACAGCAG<br>CTGAGCGATATCACCCTGATCAAGGCTGGGGCAAGCCGAGCCATCGAGAAGGT<br>GAACGAATGCGTGAAAAGTCAGTCACCCCGGATTAATTTCTGCGGCAACGGGAA<br>TCACATCCTGAGCCTGGTGCAGAACGCTCCTTATGGGCTGCTGTTTATCCATTTC<br>AGTTACAAGCCCACCAGCTTCAAGACTGTCCTGGTGTCACCTGGACTGTGCCTG<br>AGCGGAGATCGGGGCATTGCCCCCAAGCAGGGGTATTTCATCAAACAGAACGAC<br>TCCTGGATGTTTACAGGAAGCTCCTACTATTACCCCGAGCCTATCTCTGACAAGA<br>ATGTCGTGTTCATGAACAGCTGCTCCGTGAACTTCACCAAAGCCCCCTTCATCTA<br>CCTGAACAATAGCATCCCAAACCTGTCCGATTTCGAGGCTGAACTGTCTCTGTGG<br>TTTAAGAATCACACCAGTATTGCCCCTAACCTGACATTCAATAGCCATATCAACGC<br>TACATTTCTGGACCTGTATTACGAGATGAACGTTATCCAGGAATCTATTAAGAGTC<br>TGAACAGCGGCCGCCTGGAGGTGCTGTTCCAGGGCCCAGGCGGGTACATCCCA

	GAGGCACCTCGAGACGGACAGGCTTATGTGAGAAAGGATGGAGAGTGGGTCCT<br>GCTGTCCACCTTCCTGGGCCACCACCACCACCACCATCATAGCGCCTGGTC<br>CCACCCCCAGTTCGAGAAGTGA
NL63 (S-2P)	ATGAAGCTGTTCCTGATCCTGCTGGTGCTGCCCCTGGCCAGCTGCTTCTTTACCT<br>GTAACTCTAATGCCAACCTGAGCATGCTGCAGCTGGGCGTGCCCGACAATAGCT<br>CCACCATCGTGACAGGCCTGCTGCCTACACACTGGTTCTGCGCCAACCAGAGCA<br>CCAGCGTGTACTCCGCCAATGGCTTCTTTTATATCGATGTGGGCAACCACAGATC<br>TGCCTTTGCCCTGCACACCGGCTACTATGACGCCAACCAGTACTATATCTACGTG<br>ACAAATGAGATCGGCCTGAACGCCTCCGTGACCCTGAAGATCTGCAAGTTCTCTA<br>GGAACACCACATTCGACTTTCTGAGCAATGCCTCTAGCTCCTTCGATTGTATCGT<br>GAATCTGCTGTTTACCGAGCAGCTGGGAGCACCACTGGGAATCACCATCTCCGG<br>CGAGACAGTGAGACTGCACCTGTACAACGTGACCCGGACCTTCTACGTGCCAGC<br>AGCCTATAAGCTGACAAAGCTGAGCGTGAAGTGCTACTTCAATTATTCCTGCGTG<br>TTCAGCGTGGTGAACGCCACCGTGACAGTGAATGTGACCACACACAACGGCCGC<br>GTGGTGAATTACACAGTGTGCGACGATTGTAACGGCTATACCGACAATATCTTCA<br>GCGTGCAGCAGGATGGCAGAATCCCCAATGGCTTCCCCTTTAACAATTGGTTTCT<br>GCTGACCAACGGCAGCACACTGGTGGATGGCGTGTCCAGACTGTATCAGCCCCT<br>GAGGCTGACCTGCCTGTGGCCTGTGCCAGGCCTGAAGTCTAGCACCGGCTTCG<br>TGTACTTTAATGCCACAGGCTCCGACGTGAATTGTAACGGCTACCAGCACAACTC<br>TGTGGTGGATGTGATGCGCTATAATCTGAACTTCTCCGCCAATTCTCTGGACAAC<br>CTGAAGTCTGGCGTGATCGTGTTCAAGACCCTGCAGTACGATGTGCTGTTTTATT<br>GCAGCAACTCCTCTAGCGGCGTGCTGGACACCACAATCCCATTCGGCCCCTCCT<br>CTCAGCCTTACTACTGTTTCATCAACTCTACCATCAACACCACACACGTGAGCAC<br>ATTCGTGGGCATCCTGCCACCTACCGTGCGCGAGATCGTGGTGGCAAGGACCG<br>GCCAGTTTTACATCAATGGCTTCAAGTATTTTGACCTGGGCTTCATCGAGGCCGT<br>GAACTTCAACGTGACCACAGCCTCCGCCACCGACTTCTGGACAGTGGCCTTCGC<br>CACATTTGTGGATGTGCTGGTGAACGTGTCCGCCACCAATATCCAGAACCTGCT<br>GTACTGCGATTCTCCTTTCGAGAAGCTGCAGTGTGAGCACCTGCAGTTTGGCCT<br>GCAGGACGGCTTCTACAGCGCCAACTTTCTGGACGATAATGTGCTGCCTGAGAC<br>ATATGTGGCCCTGCCAATCTACTATCAGCACACCGATATCAATTTCACCGCCACA<br>GCCAGCTTTGGCGGCTCCTGCTACGTGTGCAAGCCACACCAGGTGAACATCAGC<br>CTGAATGGCAACACCTCCGTGTGCGTGAGAACAAGCCACTTCTCCATCAGATACA<br>TCTATAACAGGGTGAAGAGCGGCTCCCCAGGCGACAGCTCCTGGCACATCTACC<br>TGAAGTCCGGCACCTGTCCTTTCTCTTTTAGCAAGCTGAACAATTTCCAGAAGTTT<br>AAGACCATCTGCTTCTCTACAGTGGAGGTGCCAGGCAGCTGTAATTTTCCCCTGG<br>AGGCCACATGGCACTACACCAGCTATACAATCGTGGGCGCCCTGTACGTGACAT<br>GGTCTGAGGGCAACAGCATCACCGGCGTGCCCTATCCCGTGAGCGGCATCAGA<br>GAGTTCAGCAATCTGGTGCTGAACAACTGCACAAAGTACAACATCTACGATTACG<br>TGGGCACCGGCATCATCAGGTCTAGCAACCAGTCCCTGGCCGGCGGCATCACC<br>TACGTGTCCAATTCTGGCAACCTGCTGGGCTTCAAGAACGTGAGCACCGGCAAT<br>ATCTTTATCGTGACACCTTGCAATCAGCCAGACCAGGTGGCCGTGTATCAGCAGA<br>GCATCATCGGCGCCATGACAGCCGTGAACGAGTCCAGATACGGCCTGCAGAAC<br>CTGCTGCAGCTGCCAAATTTCTACTACGTGAGCAACGGCGGCAACAATTGCACC<br>ACAGCCGTGATGACCTACTCCAACTTTGGCATCTGTGCCGACGGCTCTCTGATC<br>CCTGTGAGGCCACGCAATTCCTCTGATAACGGCATCAGCGCCATCATCACAGCC<br>AATCTGTCTATCCCCAGCAACTGGACCACATCTGTGCAGGTGGAGTACCTGCAG<br>ATCACCAGCACACCTATCGTGGTGGATTGCGCCACCTACGTGTGCAATGGCAAC<br>CCAAGATGCAAGAATCTGCTGAAGCAGTACACCAGCGCCTGTAAGACAATCGAG<br>GACGCACTGAGGCTGTCCGCCCACCTGGAGACAAACGACGTGAGCTCCATGCT<br>GACCTTCGATTCCAATGCCTTTTCTCTGGCCAACGTGACCTCCTTCGGCGATTAT<br>AATCTGTCTAGCGTGCTGCCACAGCGGAACATCAGATCCTCTAGGATCGCAGGC<br>AGGAGCGCCCTGGAGGACCTGCTGTTTTCCAAGGTGGTGACCTCTGGCCTGGG<br>CACAGTGGACGTGGATTACAAGTCCTGCACCAAGGGCCTGTCTATCGCCGATCT<br>GGCCTGTGCCCAGTACTATAACGGCATCATGGTGCTGCCAGGAGTGGCAGACG<br>CAGAGCGCATGGCCATGTATACCGGCAGCCTGATCGGAGGAATGGTGCTGGGA<br>GGACTGACATCCGCCGCAGCAATCCCTTTCTCTCTGGCCCTGCAGGCCCGGCTG

	AATTACGTGGCCCTGCAGACCGATGTGCTGCAGGAGAATCAGAAGATCCTGGCC<br>GCCTCTTTCAACAAGGCCATCAACAATATCGTGGCCAGCTTTAGCTCCGTGAACG<br>ATGCCATCACCCAGACAGCCGAGGCCATCCACACCGTGACAATCGCCCTGAATA<br>AGATCCAGGACGTGGTGAACCAGCAGGGCAGCGCCCTGAATCACCTGACCTCC<br>CAGCTGCGCCACAATTTCCAGGCCATCAGCAACTCCATCCAGGCCATCTACGAC<br>AGGCTGGACCCCCCACAGGCAGACCAGCAGGTGGATAGGCTGATCACCGGCCG<br>GCTGGCCGCCCTGAACGCCTTCGTGAGCCAGGTGCTGAATAAGTATACAGAGGT<br>GCGCGGCAGCCGGAGACTGGCACAGCAGAAGATCAACGAGTGCGTGAAGTCTC<br>AGAGCAACCGGTACGGCTTCTGTGGCAATGGCACCCACATCTTTAGCATCGTGA<br>ATTCCGCCCCAGACGGCCTGCTGTTCCTGCACACCGTGCTGCTGCCCACAGATT<br>ATAAGAACGTGAAGGCCTGGAGCGGCATCTGCGTGGACGGCATCTACGGCTATG<br>TGCTGCGCCAGCCTAACCTGGTGCTGTACAGCGATAATGGCGTGTTCCGCGTGA<br>CCTCCCGGATCATGTTTCAGCCCAGACTGCCTGTGCTGAGCGACTTCGTGCAGA<br>TCTATAACTGTAACGTGACATTTGTGAACATCTCCAGGGTGGAGCTGCACACCGT<br>GATCCCTGACTACGTGGATGTGAACAAGACACTGCAGGAGTTTGCCCAGAACCT<br>GCCCAAGTACGTGAAGCCTAATTTCGACCTGACCCCCTTCAACCTGACATATCTG<br>AATCTGTCTAGCGAGCTGAAGCAGCTGGAGGCCAAGACCGCCTCCCTGTTCCAG<br>ACCACAGTGGAGCTGCAGGGCCTGATCGACCAGATCAACTCTACATACGTGGAT<br>CTGAAGCTGCTGAATCGGTTTGAGAACGGATCCGGATACATCCCCGAGGCCCCC<br>AGAGATGGCCAGGCCTACGTGCGGAAGGACGGCGAGTGGGTACTGCTGAGCAC<br>ATTCCTGGGCAGATCCCTGGAGGTGCTGTTCCAGGGCCCAGGCCATCACCACCA<br>TCACCACCATCATAGCGCCTGGTCCCACCCCCAGTTCGAGAAGGGCGGCGGTA<br>GTGGAGGGGGCGGATCTGGCGGCTCAGCTTGGAGCCACCCCCAGTTCGAAAAG<br>TGA
OC43 (S-2P)	ATGTTCCTGATCCTGCTGATCAGCCTGCCTACCGCCTTTGCCGTGATCGGCGAC<br>CTGAAGTGCCCACTGGATAGCCGGACAGGCTCCCTGAACAATATCGACACCGGC<br>CCCCCTTCCATCTCTACCGCCACAGTGGATGTGACAAATGGCCTGGGCACCTAC<br>TATGTGCTGGACAGGGTGTATCTGAATACCACACTGTTCCTGAACGGCTACTATC<br>CCACCAGCGGCTCCACATACCGCAACATGGCCCTGAAGGGCACAGATAAGCTGT<br>CCACCCTGTGGTTTAAGCCACCCTTCCTGTCTGACTTTATCAATGGCATCTTCGC<br>CAAGGTGAAGAACACCAAGGTGTTTAAGGATGGCGTGATGTATTCTGAGTTCCCC<br>GCCATCACCATCGGCAGCACATTTGTGAACACCTCTTACAGCGTGGTGGTGCAG<br>CCTAGGACAATCAATTCTACCCAGGACGGCGTGAACAAGCTGCAGGGCCTGCTG<br>GAGGTGAGCGTGTGCCAGTATAATATGTGCGAGTACCCCCACACAATCTGCCAC<br>CCTAAGCTGGGCAACCACTTCAAGGAGCTGTGGCACATGGATACCGGCGTGGTG<br>TCCTGTCTGTATAAGCGCAACTTCACCTACGACGTGAACGCCACCTATCTGTACT<br>TCCACTTTTACCAGGAGGGCGGCACATTCTATGCCTACTTTACCGATACAGGCGT<br>GGTGACCAAGTTCCTGTTCAACGTGTACCTGGGCATGGCCCTGAGCCACTACTA<br>TGTGATGCCACTGACATGCATCTCCCGGAGAGATATCGGCTTCACCCTGGAGTA<br>TTGGGTGACCCCCCTGACATCCCGGCAGTACCTGCTGGCCTTCAATCAGGACGG<br>CATCATCTTTAACGCCGTGGACTGCATGTCTGACTTCATGAGCGAGATCAAGTGT<br>AAGACACAGAGCATCGCCCCTCCAACCGGCGTGTATGAGCTGAACGGCTACACC<br>GTGCAGCCCATCGCCGACGTGTACAGGCGCAAGCCAGATCTGCCCAACTGCAAT<br>ATCGAGGCCTGGCTGAATGACAAGTCCGTGCCCTCTCCTCTGAACTGGGAGAGA<br>AAGACCTTCAGCAACTGTAACTTCAACATGAGCTCCCTGATGTCTTTCATCCAGG<br>CCGACAGCTTTACATGCAACAATATCGATGCCGCCAAGATCTACGGCATGTGCTT<br>CTCTAGCATCACCATCGACAAGTTTGCCATCCCTAACGGCCGGAAGGTGGATCT<br>GCAGCTGGGCAATCTGGGCTATCTGCAGTCTTTCAACTACAGAATCGATACCACA<br>GCCACAAGCTGCCAGCTGTACTATAATCTGCCAGCCGCCAACGTGTCCGTGTCT<br>CGGTTCAATCCCTCCACCTGGAACAAGAGATTCGGCTTTATCGAGAATAGCGTGT<br>TCAAGCCACAGCCAGCAGGCGTGCTGACAAACCACGACGTGGTGTACGCCCAG<br>CACTGTTTCAAGGCCCCAAAGAATTTTTGCCCCTGTAAGCTGAACTCCTCTCTGT<br>GCGTGGGCTCCGGACCAGGCAAGAACAATGGCATCGGCACCTGTCCCGCCGGC<br>ACAAACTATCTGACCTGCCACAACCTGTGCAACCCTGATCCAATCACCTTCACAG<br>GCCCCTACAAGTGCCCTCAGACCAAGAGCCTGGTGGGAATCGGAGAGCACTGC<br>TCCGGACTGGCCGTGAAGTCTGACTACTGTGGCGGCAATCCTTGCACATGTCAG

	CCACAGGCCTTCCTGGGATGGAGCGCCGACTCCTGCCTGCAGGGCGATAAGTG<br>TAATATCTTTGCCAACCTGATCCTGCACGATGTGAACTCTGGCCTGACCTGCAGC<br>ACAGACCTGCAGAAGGCCAATACCGATATCAAGCTGGGCGTGTGCGTGAACTAT<br>GACCTGTATGGCATCTCCGGCCAGGGCATCTTCGTGGAAGTGAATGCCACATAC<br>TATAATTCCTGGCAGAACCTGCTGTACGACTCTAATGGCAACCTGTATGGCTTCA<br>GGGATTACATCACCAATCGGACCTTCATGATCAGATCTTGCTATAGCGGACGCGT<br>GAGCGCCGCATTCCACGCAAACAGCTCCGAGCCTGCCCTGCTGTTCAGGAATAT<br>CAAGTGCAACTACGTGTTTAACAATTCCCTGATCCGCCAGCTGCAGCCAATCAAC<br>TATTTTGATTCTTACCTGGGCTGCGTGGTGAATGCCTACAACAGCACCGCCATCT<br>CTGTGCAGACATGCGACCTGACCGTGGGCTCCGGCTATTGCGTGGATTACTCCA<br>AGAATCGGAGATCTAGGCGCGCCATCACCACAGGCTACAGGTTCACAAACTTTG<br>AGCCTTTCACCGTGAATAGCGTGAACGACTCCCTGGAGCCTGTGGGCGGCCTGT<br>ATGAGATCCAGATCCCAAGCGAGTTCACCATCGGCAACATGGAGGAGTTTATCC<br>AGACATCTAGCCCAAAGGTGACCATCGACTGCGCAGCCTTCGTGTGCGGCGATT<br>ATGCCGCCTGCAAGTCTCAGCTGGTGGAGTACGGCAGCTTTTGTGATAATATCAA<br>CGCCATCCTGACCGAAGTGAATGAGCTGCTGGACACCACACAGCTGCAGGTGG<br>CCAATAGCCTGATGAACGGCGTGACCCTGTCCACAAAGCTGAAGGACGGCGTGA<br>ATTTCAACGTGGACGATATCAACTTTTCCTCTGTGCTGGGCTGCCTGGGCTCCGA<br>GTGTTCTAAGGCAAGCTCCCGGAGCGCCATCGAGGACCTGCTGTTCGATAAGGT<br>GAAGCTGTCCGATGTGGGCTTTGTGGCCGCCTATAACAATTGCACCGGCGGCGC<br>CGAGATCAGAGACCTGATCTGCGTGCAGAGCTACAAGGGCATCAAGGTGCTGCC<br>CCCTCTGCTGAGCGAGAACCAGATCTCCGGCTATACACTGGCAGCCACCAGCGC<br>CTCCCTGTTCCCACCATGGACAGCAGCAGCAGGCGTGCCCTTTTATCTGAATGT<br>GCAGTACCGCATCAACGGCCTGGGCGTGACCATGGACGTGCTGTCCCAGAATCA<br>GAAGCTGATCGCCAACGCCTTCAACAATGCCCTGGACGCCATCCAGGAGGGCTT<br>TGATGCCACCAATAGCGCCCTGGTGAAGATCCAGGCCGTGGTGAATGCCAACGC<br>CGAGGCCCTGAACAATCTGCTGCAGCAGCTGTCCAACCGGTTCGGCGCCATCTC<br>TAGCTCCCTGCAGGAGATCCTGTCTAGACTGGACCCTCCAGAGGCCGAGGCCCA<br>GATCGATAGGCTGATCAATGGCCGCCTGACAGCCCTGAACGCCTACGTGAGCCA<br>GCAGCTGTCTGATAGCACCCTGGTGAAGTTCTCCGCCGCCCAGGCCATGGAGAA<br>AGTGAATGAGTGCGTGAAGTCTCAGTCTAGCCGGATCAACTTTTGTGGCAATGG<br>CAACCACATCATCAGCCTGGTGCAGAACGCCCCATATGGCCTGTACTTTATCCAC<br>TTCTCTTATGTGCCCACAAAGTACGTGACCGCCAAGGTGAGCCCTGGACTGTGC<br>ATCGCAGGCGACAGAGGAATCGCACCAAAGAGCGGCTATTTCGTGAATGTGAAC<br>AATACATGGATGTACACCGGCTCCGGCTACTATTACCCCGAGCCTATCACCGAG<br>AACAATGTGGTGGTCATGTCCACATGTGCCGTGAACTATACCAAGGCCCCCTAC<br>GTGATGCTGAATACCTCTACACCTAACCTGCCAGACTTCAGGGAGGAGCTGGAT<br>CAGTGGTTTAAGAATCAGACAAGCGTGGCCCCTGACCTGTCCCTGGATTACATCA<br>ACGTGACCTTTCTGGACCTGCAGGTGGAGATGAATCGCCTGCAGGAGGCCATCA<br>AGGTGCTGAACGGATCCGGATACATCCCCGAGGCCCCCAGAGATGGCCAGGCC<br>TACGTGCGGAAGGACGGCGAGTGGGTACTGCTGAGCACATTCCTGGGCAGATC<br>CCTGGAGGTGCTGTTCCAGGGCCCAGGCCATCACCACCATCACCACCATCATAG<br>CGCCTGGTCCCACCCCCAGTTCGAGAAGGGCGGCGGTAGTGGAGGGGGCGGA<br>TCTGGCGGCTCAGCTTGGAGCCACCCCCAGTTCGAAAAGTGA
WIV-1 (S-2P)	ATGAAACTGCTGGTGCTGGTCTTTGCCACACTGGTCTCATCCTACACTATTGAAA<br>AATGCCTGGACTTTGACGACAGAACTCCCCCCGCCAACACCCAGTTTCTGAGCT<br>CCCACCGGGGCGTGTACTATCCAGACGATATCTTCAGATCCAACGTGCTGCACC<br>TGGTGCAGGATCACTTCCTGCCCTTTGACTCTAATGTGACAAGATTCATCACCTTT<br>GGCCTGAATTTCGACAACCCTATCATCCCATTCAAGGATGGCATCTACTTTGCCG<br>CCACAGAGAAGTCTAACGTGATCCGGGGCTGGGTGTTTGGCAGCACCATGAACA<br>ATAAGTCTCAGAGCGTGATCATCATGAACAATAGCACCAACCTGGTCATCAGAGC<br>CTGCAATTTCGAGCTGTGCGACAACCCCTTCTTTGTGGTGCTGAAGTCCAACAAT<br>ACACAGATCCCTTCTTATATCTTTAACAATGCCTTCAACTGCACCTTTGAGTACGT<br>GAGCAAAGACTTCAACCTGGACCTGGGCGAGAAGCCAGGCAACTTCAAGGACCT<br>GCGGGAGTTCGTGTTTAGAAACAAGGATGGCTTCCTGCACGTGTATTCCGGCTA<br>CCAGCCAATCTCCGCCGCATCTGGCCTGCCAACAGGCTTCAATGCCCTGAAGCC

	CATCTTTAAGCTGCCTCTGGGCATCAATATCACCAACTTCAGGACACTGCTGACC<br>GCATTTCCACCTCGCCCCGACTATTGGGGCACAAGCGCCGCAGCCTATTTCGTG<br>GGCTACCTGAAGCCTACCACCTTCATGCTGAAGTACGATGAGAACGGCACAATC<br>ACCGACGCCGTGGATTGCTCCCAGAATCCTCTGGCCGAGCTGAAGTGTAGCGTG<br>AAGTCCTTTGAGATCGACAAGGGCATCTATCAGACCAGCAACTTTCGGGTGGCC<br>CCATCCAAGGAGGTGGTGAGATTCCCTAATATCACAAACCTGTGCCCATTCGGC<br>GAGGTGTTTAATGCCACCACATTCCCCAGCGTGTACGCCTGGGAGCGGAAGAGA<br>ATCTCTAACTGCGTGGCCGATTATAGCGTGCTGTACAATTCCACATCTTTCAGCA<br>CCTTTAAGTGCTATGGCGTGTCCGCCACCAAGCTGAATGACCTGTGCTTCTCTAA<br>CGTGTACGCCGATAGCTTTGTGGTGAAGGGCGACGATGTGAGGCAGATCGCAC<br>CTGGACAGACCGGCGTGATCGCAGACTACAACTATAAGCTGCCAGACGATTTCA<br>CAGGCTGCGTGCTGGCCTGGAATACCCGCAACATCGATGCCACACAGACCGGC<br>AACTACAATTATAAGTACAGGAGCCTGAGGCACGGCAAGCTGAGGCCTTTCGAG<br>CGCGACATCTCCAACGTGCCCTTTTCTCCTGATGGCAAGCCTTGCACCCCACCC<br>GCCTTTAATTGTTACTGGCCACTGAACGACTATGGCTTCTACATCACAAATGGCA<br>TCGGCTATCAGCCATACAGGGTGGTGGTGCTGAGCTTCGAGCTGCTGAACGCAC<br>CAGCAACAGTGTGCGGACCAAAGCTGTCCACCGATCTGATCAAGAATCAGTGCG<br>TGAACTTCAACTTCAACGGCCTGACAGGCACCGGCGTGCTGACCCCCTCTAGCA<br>AGAGGTTCCAGCCTTTTCAGCAGTTCGGCCGCGACGTGAGCGATTTCACAGACT<br>CCGTGCGCGACCCTAAGACCTCCGAGATCCTGGATATCTCTCCATGCAGCTTTG<br>GCGGCGTGTCTGTGATCACCCCCGGCACAAACACCTCCTCTGAGGTGGCCGTG<br>CTGTACCAGGATGTGAATTGTACAGACGTGCCAGTGGCAATCCACGCAGACCAG<br>CTGACCCCTTCTTGGCGGGTGCACAGCACAGGCAACAACGTGTTCCAGACCCAG<br>GCAGGATGCCTGATCGGAGCAGAGCACGTGGATACCAGCTATGAGTGCGACATC<br>CCTATCGGCGCCGGCATCTGTGCCTCCTACCACACAGTGAGCTCCCTGAGATCC<br>ACCTCTCAGAAGTCCATCGTGGCCTATACCATGTCTCTGGGCGCCGATTCTAGCA<br>TCGCCTACAGCAACAATACAATCGCCATCCCAACCAACTTTAGCATCTCCATCAC<br>CACAGAAGTGATGCCCGTGTCCATGGCCAAGACATCTGTGGACTGCAACATGTA<br>TATCTGTGGCGATTCCACCGAGTGCGCCAATCTGCTGCTGCAGTACGGCTCCTT<br>CTGTACACAGCTGAACAGGGCCCTGTCTGGAATCGCAGTGGAGCAGGACCGGA<br>ATACCAGAGAGGTGTTCGCCCAGGTGAAGCAGATGTACAAGACACCTACCCTGA<br>AGGACTTTGGCGGCTTCAACTTTTCTCAGATCCTGCCAGATCCCCTGAAGCCAAC<br>AAAGCGGAGCTTCATCGAGGACCTGCTGTTTAATAAGGTGACCCTGGCCGATGC<br>CGGCTTCATGAAGCAGTATGGCGAGTGCCTGGGCGATATCAACGCCCGGGACC<br>TGATCTGTGCCCAGAAGTTTAATGGCCTGACAGTGCTGCCTCCACTGCTGACCG<br>ACGATATGATCGCAGCATACACAGCCGCCCTGGTGAGCGGCACAGCAACCGCA<br>GGATGGACCTTTGGCGCAGGAGCCGCCCTGCAGATCCCCTTCGCCATGCAGAT<br>GGCCTATCGGTTTAACGGCATCGGCGTGACCCAGAATGTGCTGTACGAGAACCA<br>GAAGCAGATCGCCAATCAGTTCAACAAGGCCATCAGCCAGATCCAGGAGTCCCT<br>GACCACAACCTCTACCGCCCTGGGCAAGCTGCAGGACGTGGTGAATCAGAACG<br>CCCAGGCCCTGAATACACTGGTGAAGCAGCTGTCCTCTAACTTCGGCGCCATCA<br>GCTCCGTGCTGAATGATATCCTGTCCAGACTGGACCCCCCTGAGGCAGAGGTGC<br>AGATCGATAGGCTGATCACCGGCCGCCTGCAGTCTCTGCAGACATATGTGACCC<br>AGCAGCTGATCAGGGCAGCAGAGATCAGAGCAAGCGCCAACCTGGCCGCCACA<br>AAGATGAGCGAGTGCGTGCTGGGCCAGTCCAAGAGGGTGGACTTCTGTGGCAA<br>GGGCTATCACCTGATGTCCTTTCCTCAGGCAGCACCACACGGAGTGGTGTTCCT<br>GCACGTGACCTACGTGCCATCTCAGGAGCGCAACTTTACAACCGCACCAGCAAT<br>CTGCCACGAGGGCAAGGCCTACTTCCCCAGGGAGGGCGTGTTCGTGTTTAACG<br>GCACAAGCTGGTTTATCACCCAGCGCAATTTCTTTTCCCCTCAGATCATCACAAC<br>CGACAATACATTCGTGTCTGGCAGCTGTGACGTGGTCATCGGCATCATCAACAAT<br>ACCGTGTATGATCCACTGCAGCCCGAGCTGGACTCTTTCAAGGAGGAGCTGGAT<br>AAGTACTTTAAGAACCACACCAGCCCCGACGTGGATCTGGGCGACATCAGCGGC<br>ATCAATGCCTCCGTGGTGAACATCCAGAAGGAGATCGATCGCCTGAATGAGGTG<br>GCTAAGAACCTGAATGAGTCTCTGATTGACCTGCAGGAACTGGGCAAGTATGAA<br>CAGGGATCCGGATACATCCCCGAGGCCCCCAGAGATGGCCAGGCCTACGTGCG<br>GAAGGACGGCGAGTGGGTACTGCTGAGCACATTCCTGGGCAGATCCCTGGAGG

	TGCTGTTCCAGGGCCCAGGCCATCACCACCATCACCACCATCATAGCGCCTGGT<br>CCCACCCCCAGTTCGAGAAGGGCGGCGGTAGTGGAGGGGGCGGATCTGGCGG<br>CTCAGCTTGGAGCCACCCCCAGTTCGAAAAGTGA
FcαR	GAATTCGCCACCATGGACCCCAAGCAGACCACCCTGCTGTGCCTGGTGCTGTGT<br>CTGGGCCAGAGAATCCAGGCCCAGGAAGGCGACTTCCCCATGCCCTTCATCAGC<br>GCCAAGAGCAGCCCCGTGATCCCCCTGGATGGCAGCGTGAAGATCCAGTGCCA<br>GGCCATCAGAGAGGCCTACCTGACCCAGCTGATGATCATTAAGAACAGCACCTA<br>CCGCGAGATCGGCAGACGGCTGAAGTTCTGGAACGAGACAGACCCCGAGTTCG<br>TGATCGACCACATGGACGCCAACAAGGCCGGCAGATACCAGTGTCAGTACCGGA<br>TCGGCCACTACCGGTTCCGGTACAGCGACACCCTGGAACTGGTCGTGACCGGC<br>CTGTACGGCAAGCCTTTCCTGAGCGCCGATCGGGGACTGGTGCTGATGCCCGG<br>CGAGAACATCAGCCTGACCTGTAGCAGCGCCCACATCCCCTTCGACAGATTCAG<br>CCTGGCCAAAGAGGGCGAGCTGAGCCTGCCTCAGCATCAGTCTGGCGAGCACC<br>CCGCCAACTTTAGCCTGGGCCCTGTGGACCTGAACGTGTCCGGCATCTACCGGT<br>GCTACGGCTGGTACAACCGGTCCCCCTACCTGTGGTCCTTCCCCAGCAACGCTC<br>TGGAACTGGTCGTGACAGACAGCATCCACCAGGACTACACCACCCAGAATGGCG<br>GCGGAGGATCTGGCGGAGGCGGAAGTGGCGGAGGGGGCTCTGGACTGAACGA<br>CATCTTCGAGGCCCAGAAAATCGAGTGGCACGAGCACCACCACCATCACCACTG<br>ACTCGAG
Fusion peptide	LCBiot-SKPSKRSFIEDLLFNKVTLADAGFIKQYGD

Supplementary Table 4. Sample Dilutions in the Fc Array Assay

Sample Type	Dilution	Detection Reagent
Serum	1:5000	anti-human IgG, FcγR2A, FcγR2B, FcγR3A, FcγR3B
Serum	1:1000	anti-human IgG1
Serum	1:250	anti-human IgA, anti-human IgA1, anti-human IgA2, anti-human IgD, anti-human IgM, anti-human IgG2 anti-human IgG3, anti-human IgG4, FcαR
Nasal	1:10	All detections
Stool	1:10	All detections
